# Chemically monoubiquitinated PEX5 binds to the components of the peroxisomal docking and export machinery

**DOI:** 10.1038/s41598-018-34200-5

**Published:** 2018-10-30

**Authors:** Vera Hagmann, Stefanie Sommer, Patrick Fabian, Jan Bierlmeier, Nadine van Treel, Henning D. Mootz, Dirk Schwarzer, Jorge E. Azevedo, Gabriele Dodt

**Affiliations:** 1 Interfakultäres Institut für Biochemie (IFIB), Universität Tübingen, Tübingen, Germany; 2 Institut für Biochemie, Universität Münster, Münster, Germany; 3 Instituto de Investigação e Inovação em Saúde (I3S), Instituto de Biologia Molecular e Celular (IBMC), and Instituto de Ciências Biomédicas Abel Salazar (ICBAS), University of Porto, Porto, Portugal

## Abstract

Peroxisomal matrix proteins contain either a peroxisomal targeting sequence 1 (PTS1) or a PTS2 that are recognized by the import receptors PEX5 and PEX7, respectively. PEX5 transports the PTS1 proteins and the PEX7/PTS2 complex to the docking translocation module (DTM) at the peroxisomal membrane. After cargo release PEX5 is monoubiquitinated and extracted from the peroxisomal membrane by the receptor export machinery (REM) comprising PEX26 and the AAA ATPases PEX1 and PEX6. Here, we investigated the protein interactions of monoubiquitinated PEX5 with the docking proteins PEX13, PEX14 and the REM. “Click” chemistry was used to synthesise monoubiquitinated recombinant PEX5. We found that monoubiquitinated PEX5 binds the PEX7/PTS2 complex and restores PTS2 protein import *in vivo* in ΔPEX5 fibroblasts. *In vitro* pull-down assays revealed an interaction of recombinant PEX5 and monoubiquitinated PEX5 with PEX13, PEX14 and with the REM components PEX1, PEX6 and PEX26. The interactions with the docking proteins were independent of the PEX5 ubiquitination status whereas the interactions with the REM components were increased when PEX5 is ubiquitinated.

## Introduction

Mammalian peroxisomes are single membrane-bound organelles that do not contain DNA or RNA. All matrix proteins are nuclear encoded and translated on free ribosomes and hence all these proteins must be posttranslationally imported into peroxisomes^1^. Their correct sorting to the organelle is ensured by peroxisomal targeting signals (PTS), small peptide sequences present in their primary structure that are recognized by shuttling receptors. There are two types of PTSs, the so-called PTS1 and PTS2. The PTS1^2,3^ is a short C-terminal signal peptide that is used by most peroxisomal matrix proteins. PTS1 proteins are recognized by the shuttling receptor PEX5 while still in the cytosol. This interaction involves the PTS1 itself, on one side, and the C-terminal tetratricopeptide repeat (TPR) domain of PEX5^4,5^, on the other side. The PTS2 is a degenerated nonapeptide present at the N-terminus of only a few mammalian enzymes^6,7^. PTS2 proteins are also transported to the peroxisome by PEX5. However, in this case the cargo protein-PEX5 interaction requires an extra factor, the co-receptor PEX7^8–10^. We note that mammalian PEX5 is expressed in at least two forms, PEX5L and PEX5S, which are generated by alternative splicing of the *PEX5* transcript^11,12^. PEX5L contains an insert of 37 amino acids that is positioned after amino acids 214 of PEX5S. This insert contains part of the binding-site for the PEX7/PTS2 cargo complex and thus only PEX5L (hereafter referred to as simply PEX5) can transport PTS2 proteins to the peroxisome^13,14^.

After binding their cargos in the cytosol, PEX5 or PEX5/PEX7 interact with the peroxisomal membrane docking translocation module (DTM), which in mammals comprises the peroxins 14 and 13^15^ and the three RING (really interesting new gene) finger proteins PEX2, PEX10, and PEX12^16^. The N-terminal disordered region of PEX5^17^ harbours several binding motifs for PEX14 and PEX13 that have been extensively studied by several groups^13,18–21^. The interaction process may involve partly cooperative and sequential steps^22^. Although the composition of the DTM has been analysed in detail, the stoichiometry of each of its components is still unclear and might vary under different situations^16^. PEX14 is probably the main binding site at the DTM for receptor-cargo complexes^23^. In agreement with this, PEX14 seems to interact better with cargo-loaded receptors, at least *in vitro*, whereas PEX13 probably interacts better with cargo-free PEX5. This property led some authors to suggest that PEX13 might play a role in PTS1 cargo-release from PEX5. Interestingly, the PEX5/PEX7/PTS2 protein complex can still bind to PEX13, suggesting that the mechanism of cargo-release for PTS1 and PTS2 proteins might be different^24^ (for a review on the structural aspects of these recognition processes see^22^).

After cargo release, PEX5 is monoubiquitinated at a conserved cysteine residue (cys 11 in human PEX5)^25,26^. The E3 ubiquitin ligases catalysing this reaction are the DTM components PEX2, PEX10, and PEX12 (for review see^27^). Monoubiquitinated PEX5 is then extracted from the DTM in an ATP-dependent process^28–31^ by the receptor export machinery (REM), which in mammals comprises PEX1, PEX6^29^ and PEX26^32–34^. PEX1 and PEX6 are two members of the AAA ATPases (ATPases associated with diverse cellular activities) family. Recent structural data on the yeast peroxins revealed that they form a heterohexameric complex that binds PEX15, the orthologue of mammalian PEX26^35–40^. PEX26 is a tail-anchored protein of the peroxisomal membrane and thus is thought to anchor the AAA ATPases at or near the DTM. Finally, after extraction from the DTM, monoubiquitinated PEX5 undergoes deubiquitination in the cytosol. A new protein transport cycle can then start^41^.

Although our knowledge on how proteins are sorted to the peroxisomal matrix is rather comprehensive, there are still many mechanistic aspects of this pathway that remain poorly defined. An important one regards the extraction step of PEX5 from the DTM. It is known for long that monoubiquitination of PEX5 at the DTM is mandatory for its extraction into the cytosol but exactly how PEX5-linked ubiquitin is released from the DTM by the PEX1-PEX6 heterohexamer is unknown. A major problem in addressing this issue is related to the fact that pure monoubiquitinated PEX5 suitable for protein-protein interaction studies has not been available thus far. Here, we describe the chemical synthesis of such a protein. Using a copper(I)-catalysed alkyne-azide cycloaddition (CuAAC, also known as “click” chemistry), a strategy frequently employed to produce proteins modified with ubiquitin and ubiquitin-like proteins^42–44^, we have generated large amounts of recombinant human PEX5 possessing a single ubiquitin molecule covalently attached to its residue 11.

We show that this monoubiquitinated PEX5 protein is functional in PTS2 import *in vivo* and *in vitro* and interacts with several components of the DTM and the REM *in vitro*. Importantly, monoubiquitinated PEX5 binds better to the REM components than the non-ubiquitinated PEX5 protein. Finally, our data also suggest that PEX26 interacts with PEX14 and weakly with PEX13, thus raising the possibility that PEX26 might be a part of the DTM, at least transiently, when monoubiquitinated PEX5 is exported back into the cytosol.

## Results

### Synthesis of monoubiquitinated PEX5 using the copper(I)-catalysed alkyne-azide cycloaddition (CuAAC)

After delivering its cargos to the peroxisome matrix PEX5 gets monoubiquitinated at cysteine 11, a mandatory modification for its subsequent extraction from the DTM by the receptor export machinery REM. To gain more insights in the interaction of monoubiquitinated PEX5 with the components of the export machinery, it was necessary to have suitable amounts of the pure compound. Considering that naturally occurring monoubiquitinated PEX5 is a labile and a very low abundance protein we decided to chemically synthesise large amounts of it by linking ubiquitin to recombinant human PEX5^11^ using a CuAAC reaction. A brief description of the procedure used is provided below.

**Figure 1:**
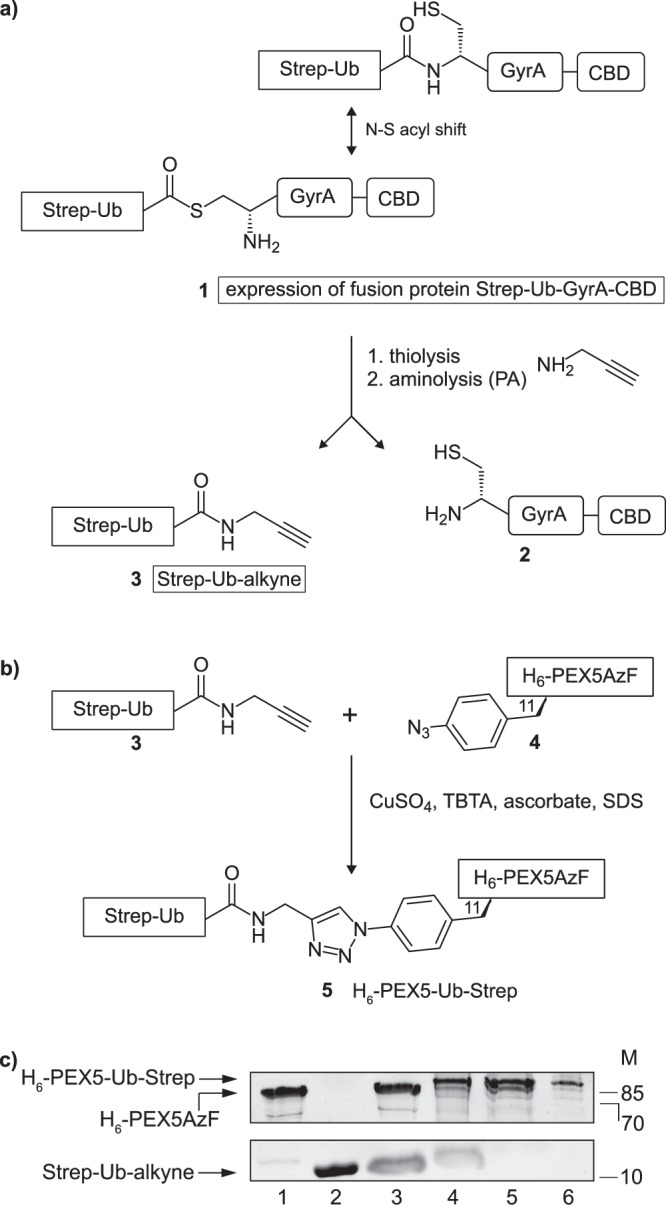
Schematic overview on the generation of H_6_-PEX5-Ub-Strep. (**a**) The expressed fusion protein Strep-Ub-GyrA-CBD (**1**) was bound to a chitin matrix by its CBD and incubated with 2-mercaptoethanesulfonic acid (MESNA) to release the Strep-Ub-MESNA thioester. Treatment of this thioester with propargylamine (PA) resulted in the Strep-Ub-alkyne (**3**). (**b**) A CuAAC reaction was performed with purified Strep-Ub-alkyne (**3**) and H_6_-PEX5AzF (**4**). The proteins were mixed together at a ratio of 3:1 in the presence of CuSO_4_, TBTA, ascorbate and SDS resulting in a stable H_6_-PEX5-Ub-Strep (**5**). (**c**) Analysis of the purification and click reaction steps of H_6_-PEX5AzF (**4**), Strep-Ub-alkyne (**3**) and H_6_-PEX5-Ub-Strep (**5**) on a 17% SDS-gel: (**4**) after Ni-column (lane 1), (**3**) after SEC (lane 2), control of CuAAC reaction (lane 3), CuAAC reaction of (**3**) and (**4**) resulting in (**5**) (lane 4). (**5**) was purified twice using a Ni-column to eliminate (**3**) (lane 5) and a *Strep*-Tactin®-column to remove (**4**) (lane 6). The arrows to the left indicate H_6_-PEX5-Ub-Strep (82 kDa), H_6_-PEX5AzF (72 kDa) and Strep-Ub-alkyne (11 kDa). The SDS-gels were stained with coomassie brilliant blue. Numbers to the right indicate the molecular mass (M) of proteins in kDa. The cropped images originate from one gel. The full-length gel is presented in Supplementary Fig. S6. GyrA = intein from *Mycobacterium xenopi*, CBD = chitin binding domain, AzF = p-azidophenylalanine, TBTA = tris(benzyltriazolylmethyl)amine.

We first produced a PEX5-azide by incorporating the unnatural amino acid p-azidophenylalanine (AzF) at position 11 of its polypeptide chain. This resulted in H_6_-PEX5C11AzF, from here on named H_6_-PEX5AzF (**4**, see Fig. 1b), which was then purified (Fig. 1c, lane 1).

To generate a ubiquitin-alkyne we generated a fusion protein (**1**) comprising (from the N- to the C-terminus) a Strep-tag, yeast ubiquitin, an intein (GyrA from *Mycobacterium xenopi)* and a chitin binding domain (CBD). Note that the ubiquitin moiety in this fusion protein lacks the last two glycines in order to approximate the native spacing between PEX5 and ubiquitin after the formation of a 1,2,3-triazole linkage by the CuAAC reaction (**5**)^42^ (see Supplementary Fig. S1 for the comparison of the native and chemical linkage). The fusion protein Strep-UbΔGG-GyrA-CBD, referred to as Strep-Ub-GyrA-CBD (**1**), was subjected to thiolysis by treatment with 2-mercaptoethanesulfonic acid (MESNA). Subsequent addition of propargylamine (PA) resulted in Strep-Ub-alkyne (**3**) that was then purified by size exclusion chromatography (SEC) and verified by ESI-MS (Fig. 1c, lane 2 and Supplementary Fig. S2).

Next, a CuAAC reaction was performed using purified Strep-Ub-alkyne (**3**) and H_6_-PEX5AzF (**4**) at a ratio of 3:1 (Fig. 1b). The reaction was monitored by SDS-PAGE analysis which revealed the appearance of a new species migrating 10–15 kDa above H_6_-PEX5AzF (**4**), indicating formation of the desired product H_6_-PEX5AzF-Ub-Strep, referred to as H_6_-PEX5-Ub-Strep (**5**) (Fig. 1c, compare lanes 3 and 4). Residual starting materials (*i.e*., non-clicked Strep-Ub-alkyne and H_6_-PEX5AzF) were removed by subjecting the reaction mixture to two purification steps (Fig. 1c, lanes 5 and 6; see materials and methods for details). The chemically ubiquitinated structure differs slightly in the linkage between the PEX5 and ubiquitin motif from the natural one but was expected to retain all the key interactions of ubiquitinated PEX5 (see Supplementary Fig. S1 for the comparison of the native and chemical linkage).

### H_6_-PEX5-Ub-Strep binds a PEX7/PTS2 protein complex *in vitro* and is functional in importing PTS2 proteins *in vivo*

**Figure 2:**
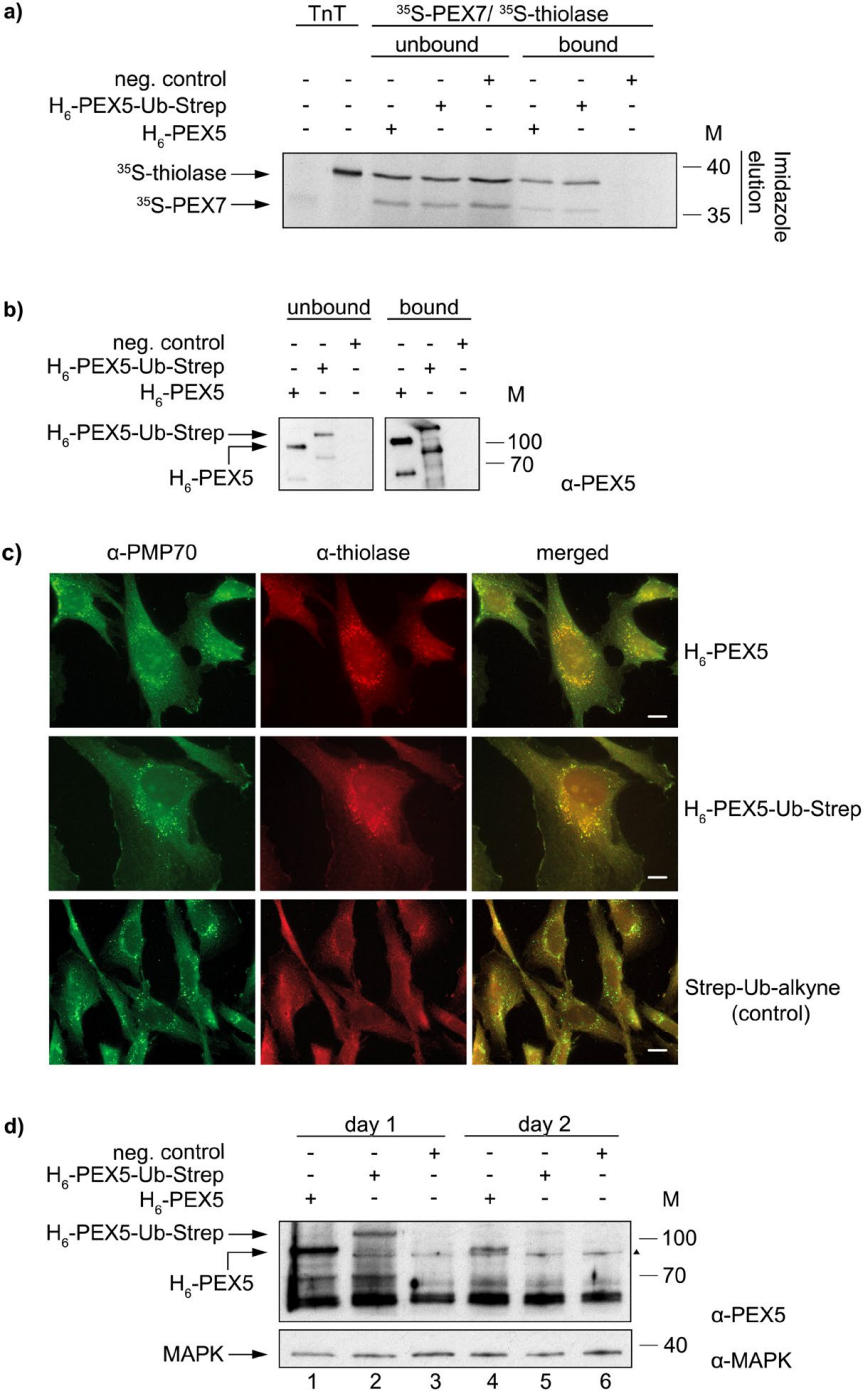
Functional studies of recombinant H_6_-PEX5-Ub-Strep *in vivo* and *in vitro*. (**a**) Pull-down assay with magnetic Ni-beads that were coupled with purified H_6_-PEX5 and H_6_-PEX5-Ub-Strep, respectively or without protein (control). After incubating the beads with *in vitro* synthesised ^35^S-PEX7, ^35^S-thiolase, buffer A and ATP, the samples were eluted with imidazole. The bound (50%) and the unbound (10%) fractions as well as the input (TNT) were analysed by SDS-PAGE/autoradiographic detection. (**b**) The bound (2.9%) and unbound (1.1%) fractions of the pull-down assay in (**a**) were also analysed by immunoblot detection with α-PEX5. The lower migrating band of PEX5 is a common degradation product in *E. coli* that also gets ubiquitinated. The two cropped blots originate from one gel. The pull-down is representative for four experiments. (**c**) Human ΔPEX5 fibroblasts were electroporated with purified H_6_-PEX5, H_6_-PEX5-Ub-Strep and Strep-Ub-alkyne (control), respectively. On the second day, an immunofluorescent staining was performed against thiolase (Alexa 594) and the peroxisomal membrane protein PMP70 (Alexa 488). Both proteins, H_6_-PEX5 and H_6_-PEX5-Ub-Strep could import thiolase into peroxisomes which is co-localised with PMP70, while no import was detected in the control. The experiment was performed twice with H_6_-PEX5-Ub-Strep and four times with H_6_-PEX5. Complementation rates were calculated by counting punctate thiolase positive cells. At least 500 cells were counted for each electroporation. The scale bar represents 20 µm. (**d**) The stability of purified H_6_-PEX5 and H_6_-PEX5-Ub-Strep after electroporation in ΔPEX5 fibroblasts was analysed after the first and the second day by immunoblot detection using α-PEX5. MAP-Kinase (MAPK) was used as a loading control and detected with α-MAPK. Both cropped blots are from one blot that has been cut horizontally, developed with the different antibodies and exposed on a single film together. The numbers to the right indicate the molecular mass (M) of proteins in kDa (**a**,**b** and **d**). The full-length versions of all gels are presented in Supplementary Fig. S6. (▲): The triangle to the right represents an unspecific protein band.

To test the functionality of the chemically synthesised H_6_-PEX5-Ub-Strep protein several experiments were performed. In one of these we asked whether H_6_-PEX5-Ub-Strep can interact with the PEX7/PTS2 protein complex *in vitro*. Figure 2a shows the results of a pull-down assay performed with magnetic Ni-beads coupled with purified H_6_-PEX5 or H_6_-PEX5-Ub-Strep or no recombinant protein (negative control). The prepared Ni-beads were incubated with ^35^S-pre-thiolase-myc (a PTS2 protein, referred to as ^35^S-thiolase) and ^35^S-myc-PEX7 (referred to as ^35^S-PEX7) which were synthesised *in vitro* in the presence of ^35^S-methionine. The results show that both H_6_-PEX5 and H_6_-PEX5-Ub-Strep, can bind the two radiolabelled proteins (Fig. 2a). Note that in contrast to ^35^S-thiolase (which contains 12 methionines) ^35^S-PEX7 contains only two methionines in its primary structure, hence the weak signal in the autoradiograph. We obtained similar results with other PTS2-reporter proteins such as PTS2-CAT (a bacterial chloramphenicol transferase) and PTS2-GFP (see Supplementary Fig. S3). Unexpectedly, similar pull-down assays using radiolabelled PTS1 proteins, such as PTS1-GFP and pre-SCP2 (pre-sterol carrier protein 2), revealed that the clicked H_6_-PEX5-Ub-Strep is not able to bind these proteins (see Supplementary Fig. S4a,c) Subsequent experiments suggest that the click chemistry conditions affect the function of the TPR domain of PEX5 (see Supplementary Fig. S4c lane 4 and 5 and discussion).

Finally, we examined the capacity of H_6_-PEX5-Ub-Strep to complement the peroxisomal protein import defect of ΔPEX5 fibroblasts. In these cells, the import of PTS1 and PTS2 proteins is impaired and therefore many of these proteins display a cytosolic localization^45,46^. For this purpose, we used electroporation to introduce purified H_6_-PEX5-Ub-Strep, H_6_-PEX5 or Strep-Ub-alkyne (control) into ΔPEX5 fibroblasts. Two days after electroporation, immunofluorescent staining was performed using antibodies against endogenous thiolase and against the peroxisomal membrane protein PMP70. As shown in Fig. 2c electroporation of both proteins, H_6_-PEX5 and H_6_-PEX5-Ub-Strep, resulted in the import of thiolase into peroxisomes as determined by its colocalization with PMP70 (Fig. 2c, “merged” panels). We note that larger complementation rates were obtained with H_6_-PEX5 (32.4%) than with H_6_-PEX5-Ub-Strep (11.3%). No complementation could be detected when Strep-Ub-alkyne was electroporated. Immunoblot analysis of lysates from cells harvested one and two days after electroporation revealed that already on the first day the amount of H_6_-PEX5-Ub-Strep was reduced compared to H_6_-PEX5 (Fig. 2d, lanes 2 and 1, respectively). Furthermore, two days after electroporation we could still detect H_6_-PEX5 in cell lysates whereas H_6_-PEX5-Ub-Strep was barely detectable (Fig. 2d, lanes 4 and 5, respectively). Apparently, although H_6_-PEX5-Ub-Strep can complement the PTS2-import defect in ΔPEX5 fibroblasts it seems to be more unstable than H_6_-PEX5 (see discussion). We also asked whether H_6_-PEX5-Ub-Strep could restore protein import of EGFP-PTS1 in ΔPEX5 fibroblasts. However, in agreement with the PTS1 protein pull-down assays described above, H_6_-PEX5-Ub-Strep did not complement the import defect of PTS1 proteins in ΔPEX5 fibroblasts while the complementation rate for H_6_-PEX5 was 14% (see Supplementary Fig. 4b and discussion).

### Recombinant H_6_-PEX5-Ub-Strep enables import of PTS2 proteins into peroxisomes *in vitro*

Next, we addressed the question whether H_6_-PEX5-Ub-Strep can be used in an *in vitro* import system using post-nuclear supernatant (PNS) from mouse liver^47,48^. To investigate the import of PTS2 proteins into peroxisomes PNS containing import-competent peroxisomes was incubated with radiolabelled ^35^S-PEX5, ^35^S-pre-thiolase and ^35^S-PEX7 and with different amounts of recombinant PEX5 proteins for 30 min, to allow import into the organelles. The PNS was subsequently treated with proteinase K to eliminate all not imported proteins.

**Figure 3:**
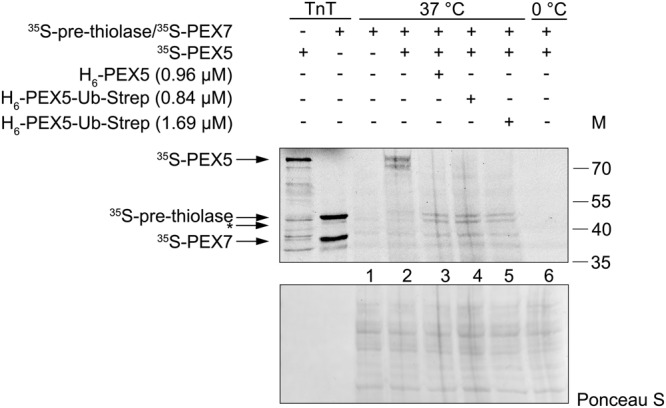
H_6_-PEX5 and H_6_-PEX5-Ub-Strep are functional in the PTS2 import into peroxisomes *in vitro*. Post-nuclear supernatant (PNS) from mouse liver was incubated with the *in vitro* ^35^S-synthesised proteins PEX5, PEX7 and pre-thiolase, and recombinant H_6_-PEX5 and H_6_-PEX5-Ub-Strep for 30 min at 37 °C or at 0 °C. After the import reaction, the samples were treated with proteinase K, precipitated and analysed by SDS-PAGE, blotting and autoradiography. The arrows to the left indicate ^35^S-PEX5, ^35^S-PEX7, and ^35^S-pre-thiolase. The processed, mature ^35^S-mat-thiolase is indicated by an asterisk (*). The Ponceau S-stained membrane served as loading control. The numbers to the right indicate the molecular mass (M) of proteins in kDa. The uncropped version of the blot is presented in Supplementary Fig. S6. The import reaction is representative for two experiments.

No protease protection of ^35^S-PEX7/^35^S-pre-thiolase could be observed when these proteins were incubated alone with PNS (Fig. 3, lane 1). This indicates that endogenous PEX5 is a limiting factor for the import of matrix proteins and that no processing of the ^35^S-pre-thiolase into the mature (mat) form occurred. A small amount of ^35^S-PEX7/^35^S-pre-thiolase is protease protected and processed when ^35^S-PEX5 was added (Fig. 3, lane 2). ^35^S-PEX5 itself also seems to be protease protected and embedded into the DTM. The addition of recombinant H_6_-PEX5 and H_6_-PEX5-Ub-Strep leads to a competition with the ^35^S-PEX5 and results in a higher amount of protease-protected and processed thiolase (Fig. 3, lanes 3 to 5). This indicates that both recombinant proteins are functional for PTS2 protein import. Increasing the amount of recombinant H_6_-PEX5-Ub-Strep does not enhance the system but leads to less import (Fig. 3, lane 5). At 0 °C neither ^35^S-PEX5 nor ^35^S-PEX7 or ^35^S-thiolase are protease-protected, indicating no import of these components (Fig. 3, lane 6).

### H_6_-PEX5-Ub-Strep interacts with the receptor export module components PEX1, PEX6 and PEX26 and with PEX14 and PEX13

**Figure 4:**
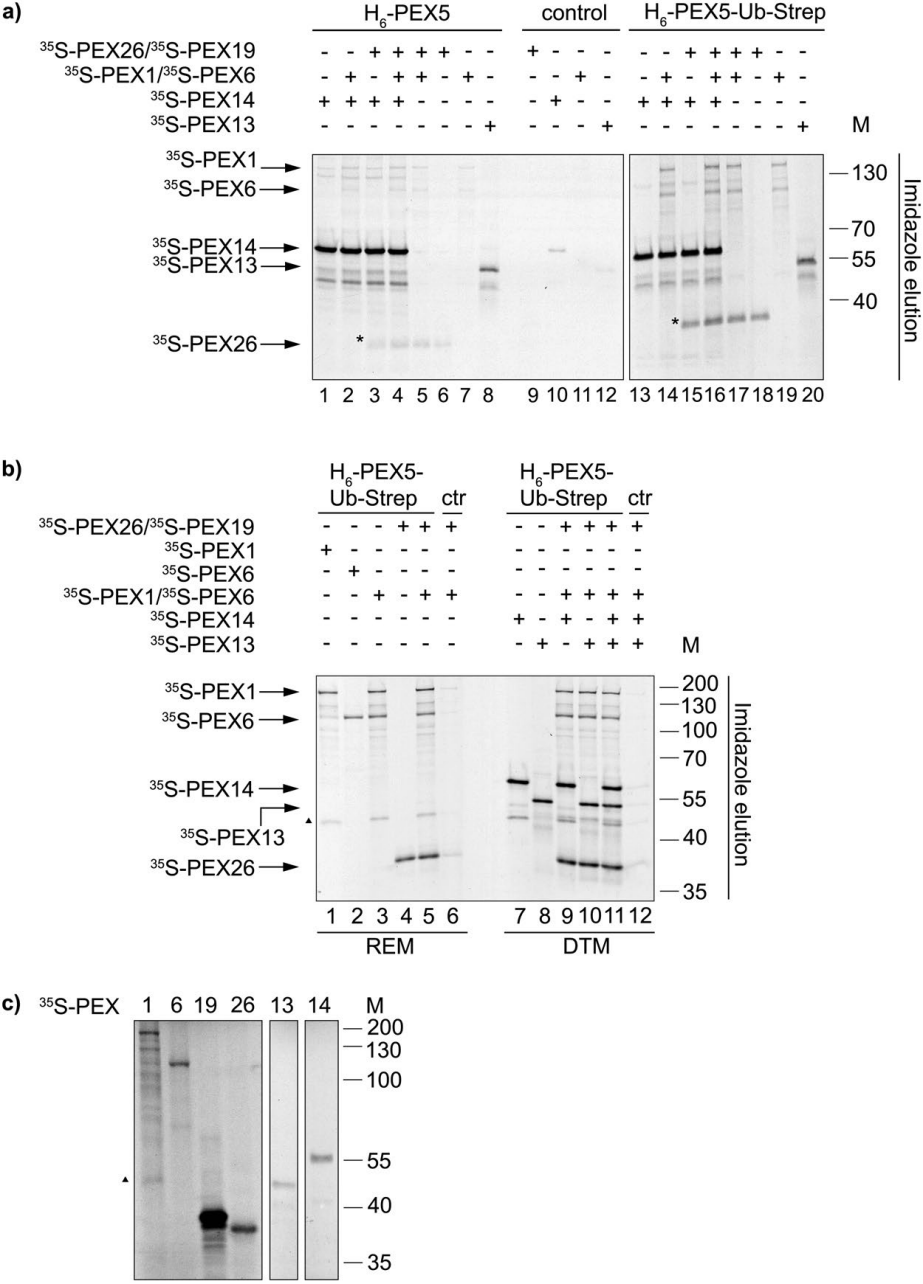
H_6_-PEX5 and H_6_-PEX5-Ub-Strep interact with the peroxisomal export machinery and with PEX13 and PEX14. (**a**) Pull-down assay with magnetic Ni-beads that were coupled with purified H_6_-PEX5, H_6_-PEX5-Ub-Strep or used uncoupled (control). *In vitro* synthesised ^35^S-PEX1/^35^S-PEX6, ^35^S-PEX26/^35^S-PEX19, ^35^S-PEX14 and ^35^S-PEX13 were added to the coupled beads and incubated with cytosol from human ΔPEX5 fibroblasts and ATP. The fractions were eluted with imidazole. The bound fractions (50%) were analysed by SDS-PAGE/autoradiography. Both gels originate from one experiment. The gels were processed in parallel. The results are representative for three individual experiments. (*): The asterisk indicates ^35^S-Pex26. (**b**) Pull-down assay with magnetic Ni-beads that were coupled with H_6_-PEX5-Ub-Strep or used uncoupled (control). *In vitro* synthesised ^35^S-PEX26/^35^S-PEX19 and REM proteins (^35^S-PEX1, ^35^S-PEX6) or DTM proteins (^35^S-PEX14, ^35^S-PEX13) were added to the beads and incubated with buffer A and ATPγS. The samples were eluted with imidazole. The bound fractions (50%) were analysed by SDS-PAGE/autoradiography. The experiments were performed twice. (**c**) *In vitro* TNT reaction of ^35^S-labelled PEX1, PEX6, PEX19, PEX26, PEX13 and PEX14 analysed by SDS-PAGE/autoradiography. The TNT reactions for PEX13 and PEX14 are from different gels. The numbers to the right indicate the molecular mass (M) of proteins in kDa (**a**–**c**). The full-length gels are presented in Supplementary Fig. S6. (▲): The triangle to the left in (**b**,**c**) represents a translation product of PEX1 that binds to H_6_-PEX5-Ub-Strep.

Export of DTM-embedded monoubiquitinated PEX5 is an ATP-dependent step catalysed by the receptor export module, which comprises PEX1, PEX6 and PEX26. Aiming to better understand the role of ubiquitin in this process, a series of *in vitro* pull-down assays were performed. We used magnetic Ni-beads containing purified H_6_-PEX5, H_6_-PEX5-Ub-Strep or no recombinant protein (negative control) as baits. *In vitro* synthesised ^35^S-PEX1 and ^35^S-PEX6 (individually or pre-mixed) and ^35^S-PEX26/^35^S-PEX19 (note that PEX26 was co-synthesised with PEX19 in order to ensure that it remains soluble; see materials and methods for details) were investigated as preys. Some of these assays also contained ^35^S-PEX14 and ^35^S-PEX13, two components of the DTM. The aim was to cover the possibility that the interactions between PEX5 and the REM components might be dependent on DTM proteins. The results of these experiments are shown in Fig. 4 and Supplementary Fig. S5a. The samples used for Fig. 4a and Supplementary Fig. S5a were incubated with ATP and cytosol from ΔPEX5 fibroblasts to provide putative co-factors that might be required for some protein-protein interactions.

Both, H_6_-PEX5 and H_6_-PEX5-Ub-Strep pulled-down ^35^S-PEX1 and ^35^S-PEX6 (Fig. 4a, lanes 7 and 19, respectively and Supplementary Fig. S5a). However, the amounts of radiolabelled PEX1 and PEX6 recovered with H_6_-PEX5-Ub-Strep were clearly larger than those obtained with H_6_-PEX5. A similar result was found with ^35^S-PEX26. Note that although large amounts of ^35^S-PEX19 were present in these assays (to maintain ^35^S-PEX26 in solution) almost no PEX19 was recovered in the pull-down samples (Fig. 4a compared to 4c, see also Supplementary Fig. S5a, lanes 1 and 5). These results strongly suggest distinct interactions of H_6_-PEX5 and H_6_-PEX5-Ub-Strep with the peroxisomal export proteins PEX1, PEX6 and PEX26.

In addition, both, H_6_-PEX5 (Fig. 4a, lanes 1 and 8, respectively) as well as H_6_-PEX5-Ub-Strep (Fig. 4a, lanes 13 and 20, respectively) have the capability to bind to the peroxisomal docking proteins ^35^S-PEX13 and ^35^S-PEX14. Interestingly, we consistently found that slightly more ^35^S-PEX26 was recovered with the H_6_-PEX5 and H_6_-PEX5-Ub-Strep beads when these assays were made in the presence of ^35^S-PEX14 and ^35^S-PEX1/^35^S-PEX6 (Fig. 4a, compare lanes 4 and 16 with lanes 6 and 18, respectively). Additional binding assays performed in binding buffer instead of cytosol from ΔPEX5 fibroblasts and in the presence of ATPγS instead of ATP yielded similar results (Fig. 4b). The eluates of the samples in Fig. 4a,b were additionally blotted for PEX5 to investigate the binding to the Ni-beads (see Supplementary Fig. 5b,c). We regularly noticed that less H_6_-PEX5-Ub-Strep compared to H_6_-PEX5 could be coupled to the Ni-beads (see Supplementary Fig. 5b). The results above suggest that ^35^S-PEX1/^35^S-PEX6, ^35^S-PEX13, ^35^S-PEX14 and ^35^S-PEX26 interact independently with H_6_-PEX5 and H_6_-PEX5-Ub-Strep.

### The interaction between PEX26 and the DTM proteins

**Figure 5:**
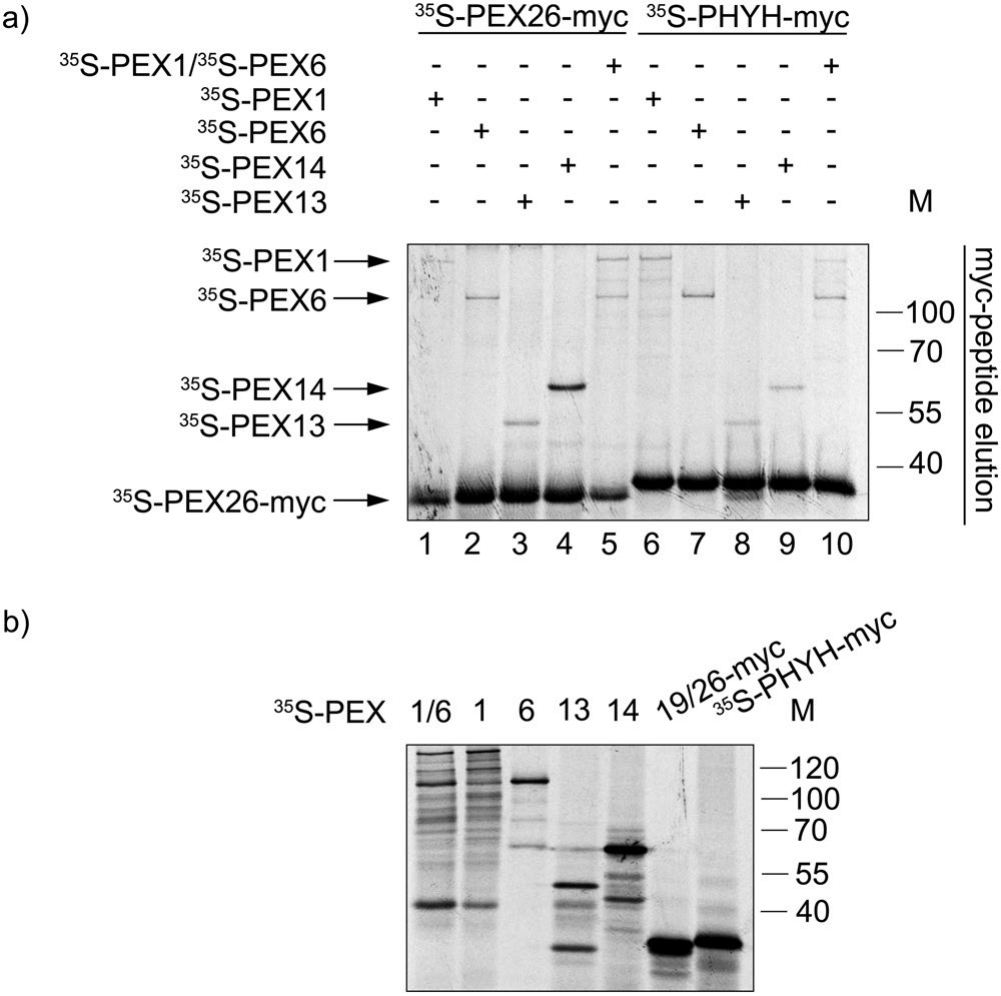
Interaction of PEX26 with the DTM proteins. Pull-down assay with PEX26-myc and the myc-tagged phythanoyl-CoA-hydroxylase (PHYH-myc) (control) with the REM and DTM proteins. (**a**) *In vitro* synthesised proteins (TNT) ^35^S-PEX26-myc/^35^S-PEX19, ^35^S-PEX1, ^35^S-PEX6, ^35^S-PEX14, ^35^S-PEX13 and ^35^S-PHYH-myc were added to magnetic myc-Trap®-beads and incubated with buffer C and ATP. The samples were eluted with myc-peptide. The bound fractions (50%) were analysed by SDS-PAGE/autoradiography. (**b**) *In vitro* TNT reaction of ^35^S-PEX26-myc/^35^S-PEX19, ^35^S-PEX1, ^35^S-PEX6, ^35^S-PEX14, ^35^S-PEX13 and ^35^S-PHYH-myc analysed by SDS-PAGE/autoradiography. The numbers to the right indicate the molecular mass (M) of proteins in kDa. The full-length gels are presented in Supplementary Fig. S6. The experiment is representative for four replicates.

To better characterize the interactions involving PEX26 and other components of the peroxisomal import machinery, additional pull-down assays were performed, but this time using as a bait *in vitro* synthesised myc-tagged ^35^S-PEX26. As shown in Fig. 5, only low amounts of radiolabelled prey proteins (PEX1, PEX6, PEX13 and PEX14) were recovered in these assays. The fact that only nanograms of bait are used in these experiments (in contrast with typical pull-down assays in which micrograms of recombinant bait proteins are generally used) may explain this result. For radiolabelled PEX1, and probably also for PEX6, the amounts recovered with PEX26-myc beads do not differ much from those appearing in the negative control with myc-tagged phythanoyl-CoA-hydroxylase (PHYH-myc) (Fig. 5, compare lanes 1 and 2 with lanes 6 and 7, respectively). However, a clear enrichment of radiolabelled PEX14 at PEX26-myc beads compared to beads loaded with the negative control was observed (Fig. 5, lanes 4 and 9). A possible PEX13 binding seems to be weak. Thus, these results suggest that PEX14 interacts with PEX26, as described previously^34,49^.

### PEX6 and PEX26 bind to H_6_-PEX5-Ub-Strep

**Figure 6:**
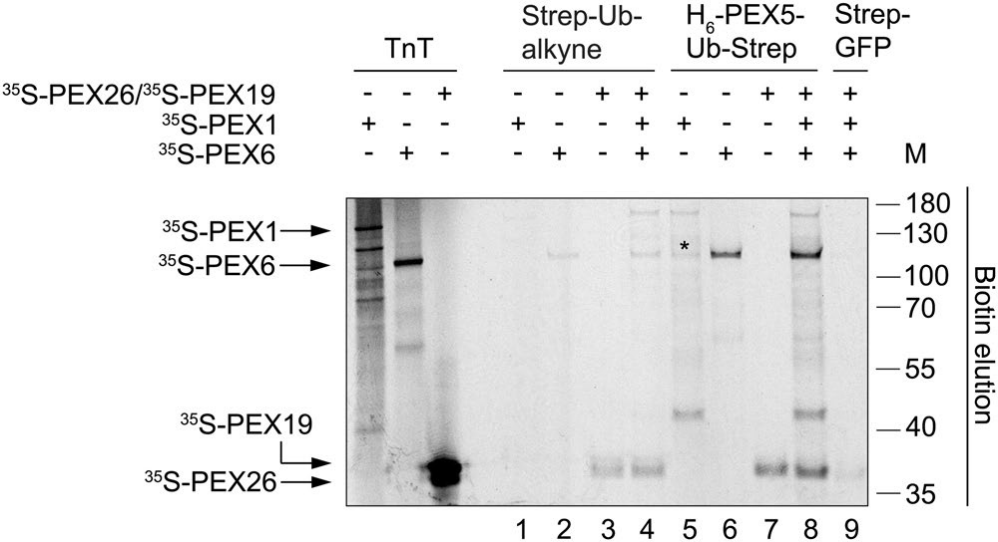
PEX6 and PEX26 bind to H_6_-PEX5-Ub-Strep. Pull-down assay with magnetic Strep-Tactin®XT-beads that were coupled with purified Strep-Ub-alkyne, H_6_-PEX5-Ub-Strep or H_6_-BRD4-GFP-Strep (referred to as Strep-GFP; control). *In vitro* synthesised ^35^S-PEX26/^35^S-PEX19, ^35^S-PEX1, ^35^S-PEX6 (TNT) were added to the beads and incubated with buffer C and ATP. The samples were eluted with buffer C containing biotin. The eluates (50%) were analysed by SDS-PAGE/autoradiography. The numbers to the right indicate the molecular mass (M) of proteins in kDa. The full-length gels are presented in Supplementary Fig. S6. The experiment is representative for three replicates. (*): The asterisk indicates a translation product of PEX1 that binds especially to H_6_-PEX5-Ub-Strep and migrates almost to the same position as PEX6.

Given the crucial role of ubiquitin in the dislocation of PEX5 from the DTM, we next asked whether PEX1, PEX6 and PEX26 can bind ubiquitin itself. For this purpose, magnetic Strep-beads containing Strep-Ub-alkyne, H_6_-PEX5-Ub-Strep or H_6_-BRD4-GFP-Strep (referred to as Strep-GFP) were used in pull-down assays with *in vitro* synthesised ^35^S-PEX1, ^35^S-PEX6, and ^35^S-PEX26/^35^S-PEX19, or a mixture of these proteins. Both, radiolabelled PEX6 and PEX26 bound much better to the H_6_-PEX5-Ub-Strep-containing beads than to those containing Strep-Ub-alkyne (Fig. 6, compare lanes 6 and 7 with lanes 2 and 3, respectively), while almost no binding occurred to the control with Strep-GFP-coupled beads. The interaction of PEX1, PEX6, and PEX26 together with Strep-Ub-alkyne is weak, compared to the stronger binding to H_6_-PEX5-Ub-Strep (see Fig. 6 lanes 4 and 8).

## Discussion

To get more insights into the extraction of monoubiquitinated PEX5 from the DTM by the REM components we generated PEX5 that was chemically ubiquitinated (H_6_-PEX5-Ub-Strep, see Fig. 1). *In vitro* analysis revealed that H_6_-PEX5-Ub-Strep binds PEX7/PTS2 proteins (Fig. 2a and Supplementary Fig. 3) and interacts with the DTM components PEX14 and PEX13 (Fig. 4). In addition, this protein is functional in PTS2 protein import as shown by complementation studies in ΔPEX5 fibroblasts with endogenous thiolase (Fig. 2c) and by the *in vitro* import assay (Fig. 3). Thus, we have no evidence that the binding of H_6_-PEX5-Ub-Strep to PEX7/PTS2 proteins or to DTM proteins is affected by the ubiquitination. This also indicates that the deubiquitination is not a mandatory step neither for the binding to cargo nor for the binding to PEX13 and PEX14.

However, both the PTS1 protein pull-down assays and the complementation studies in ΔPEX5 fibroblasts indicate that H_6_-PEX5-Ub-Strep is not able to bind to PTS1 proteins and thus cannot complement the import defect of PTS1 proteins in ΔPEX5 fibroblasts (Supplementary Fig. 4). We performed additional experiments that suggested that the oxidative conditions used in the CuAAC reaction affect the PTS1-binding domain of PEX5 (there are 5 cysteines in this domain) inducing small conformational changes. Indeed, H_6_-PEX5 subjected to this treatment (H_6_-PEX5 “click”) as a control did not interact with PTS1 proteins in an *in vitro* pull-down experiment. This was shown using the PTS1 protein pre-SCP2 as an example (Supplementary Fig. 4c). We also recognized that the electroporation of purified H_6_-PEX5-Ub-Strep into ΔPEX5 fibroblasts resulted in a significant reduction of the amount of this protein after the first and the second day (Fig. 2d). Albeit H_6_-PEX5-Ub-Strep is functional in recovering the PTS2 protein import it is conceivable that the half-life of H_6_-PEX5-Ub-Strep might be shorter than that of H_6_-PEX5. The chemically added ubiquitin cannot be cleaved from PEX5, in contrast to the wild type modification. With one ubiquitin permanently attached, it is possible that PEX5 is poly-ubiquitinated leading to an increased proteasomal degradation. We do not have any data whether H_6_-PEX5-Ub-Strep is used for several rounds of peroxisomal transport like it is assumed for the endogenous PEX5^28,50–52^ or just once. In the complementation studies in ΔPEX5 fibroblasts (Fig. 2) H_6_-PEX5-Ub-Strep was added in a vast excess to compensate for the short half-life of this protein.

Although the REM proteins were thought to be involved in the export of monoubiquitinated PEX5, until very recently neither the binding of Ub-PEX5 to the PEX1/PEX6 complex nor to PEX26 had been reported^32,34^. Our results show a binding of H_6_-PEX5 to the docking/REM components that is weak but clearly increased when PEX5 is ubiquitinated. The interactions were independent of each other (Fig. 4 and Supplementary Fig. S5). While this manuscript was under review similar findings, *i.e*. the result that the AAA ATPases Pex1p and Pex6p interact with N-terminally linked ubiquitin-Pex5p fusion protein were reported for the yeast proteins^53^.

Our *in vitro* binding assays suggest that in particular PEX6 and PEX26 proteins bind only slightly to H_6_-PEX5 (Fig. 4a and Supplementary Fig. S5a) and do not, or bind only slightly to Strep-Ub-alkyne (Fig. 6). However, both PEX6 and PEX26 independently display a substantial binding to ubiquitinated PEX5 (Fig. 4 and Supplementary Fig. S5a). A similar binding behaviour of PEX1 to H_6_-PEX5-Ub-Strep was obtained, if the latter was pulled down using the His-tag (Ni-beads) (Fig. 4). However, if H_6_-PEX5-Ub-Strep was coupled engaging the Strep-tag (Strep-beads), less PEX1 could be pulled down (Fig. 6, lane 5). This might indicate a steric hindrance for the PEX1 binding to H_6_-PEX5-Ub-Strep when the protein is coupled to the beads using the Strep-tag of the ubiquitin moiety. Thus, this would support the idea that the PEX1 binding engages the ubiquitin part of H_6_-PEX5-Ub-Strep. This has also been shown recently using a photo-crosslinking approach in an *in vitro* export assay^31^ or in an assay with purified yeast proteins^53^. Thus, it seems likely that H_6_-PEX5-Ub-Strep provides binding-sites for the docking and REM proteins. It has been shown that PEX5 comprising the first 10–125 residues can be correctly monoubiquitinated and exported from peroxisomes^17,23,31^, indicating that the binding sites are located in this first N-terminal part of PEX5. Taken together, our data may present a stalled situation where the maximum amount of interactions is possible. Especially, as we encounter almost no differences when we compare the pulldowns in the presence of ATP and cytosol (Fig. 4a) with the results with binding buffer and ATPγS (Fig. 4b). With ATPγS the action of the AAA ATPases PEX1 and PEX6 should be blocked.

We assume that PEX5 regardless of the ubiquitination status is embedded in the pore that is formed by the DTM. Given that PEX26 interacts with PEX14 and monoubiquitinated PEX5, we hypothesise that PEX26 is also located in a close distance to the DTM or is even a part of it. This assumption is additionally encouraged by quantitative proteomic studies with PEX14 indicating that yeast PEX15 is a component of the transient pore^16^. Thus, PEX26 might act as a bridge between embedded (monoubiquitinated) PEX5, the DTM and PEX1/PEX6 complex. In recent studies, *Miyata et al*. identified AWP1/ZFAND6 as a PEX5 export factor that recognizes PEX6 and monoubiquitinated PEX5 and enhances the export of PEX5 from the peroxisomal membrane^54^, but did not bind the PEX1/PEX6 complex. Our interaction studies demonstrate that the binding of ubiquitinated PEX5 and PEX6 does not require additional cytosol. However small amounts of rabbit reticulocyte lysate are present in our pull-down assays and thus we cannot exclude that traces of rabbit AWP1 or other factors are present and needed for the interactions. According to very recent data the PEX1/PEX6 complex extracts Ub-PEX5 from the peroxisomal DTM using a processive threading mechanism^31,40^. However, exactly how the PEX1/PEX6 complex engages the DTM-embedded Ub-PEX5 protein and what is the mechanistic role of ubiquitin in all this process remains completely unknown. Clearly, an answer to these questions will require reconstituting the extraction reaction *in vitro* using pure recombinant proteins. The chemical synthesis of the bona fide substrate of the PEX1/PEX6 unfoldase described here is thus the first step towards this goal.

## Methods

### Cloning

For protein production, N-terminal hexahistidinyl-tagged *hsPEX5L* was cloned into *Nco*I/*Not*I sites of pET9dNHis_6_ (a kind gift of G. Stier, Heidelberg, for a brief description see^55^) creating pVS1 (kindly provided by W. Schliebs, Bochum). Briefly, the *Nco*I/*Bgl*II fragment from pGD106^11^ containing the cDNA for PEX5L was first ligated into the *Nco*I/*BamH*I sites of pET21d+, cut with *Nco*I/*Not*I and finally cloned into pet9dNHis_6_ creating pVS1. For site-directed mutagenesis of pVS1 the forward primer (GD496) (5′-GTGGAGGCCGAATAGGGGGGTGCCACC-3′ and the reverse primer (GD467) (5′-GTTGGCACCCCCCTATTCGGCCTCCAC-3′) were used to generate the cysteine-amber stop codon mutations at position 11 resulting in *H*_6_*-PEX5C11AzF* (pVS06). To generate Strep-UbΔGG-GyrA-CBD (pVS09; referred to as Strep-Ub-GyrA-CBD) an N-terminal Strep-tag®II and a TEV-cleavage site was amplified by oligonucleotide hybridization using the forward primer (GD504) (5′-TATGTCCGCCTGGAGCCACCCGCAGTTCGAAAAAGAAAACCTGTATTTTCAGGGCCA-3′) and the reverse primer (GD505) (5′-TATGGCCCTGAAAATACAGGTTTTCTTTTTCGAACTGCGGGTGGCTCCAGGCGGACA-3′) and cloned into the *Nde*I/*Nde*I sites of pNW04^42^. The ubiquitin used in this work is derived from *S. cerevisiae*.

Full-length *hsPEX13* was amplified from cDNA using the forward primer (GD144) (5′-CAGGATCCGGTACCTTACCATGGCGTCCCAGCCGCCAC-3′) and the reverse primer (GD145) (5′-GGTCTAGATCAAAGACCTTGCTTTTCTCCATC-3′) and cloned into the *BamH*I/*Xba*I sites of pcDNA3.1/Zeo (pCK19). The PEX1 construct pBM57 contains the coding region of full-length human *hsPEX1* including 411 bp of the 3′untranslated region in the *BamH*I/*Xho*I sites of pcDNA3.1/Zeo. The PEX26-myc plasmid (pJD10) encodes *hsPEX2*6 with a C-terminal myc-(epitope) tag in the *EcoR*I/*Not*I sites of pcDNA3.1/Zeo.

### Expression in *E. coli*

*H*_*6*_*-PEX5* was expressed in *E. coli* BL21 (DE3) in 2 L of YEP (10 g peptone, 10 g NaCl and 5 g yeast extract per 1 mL) containing appropriate antibiotics. Cultures were grown to an OD_600_ = 0.4–0.6 at 37 °C, induced with IPTG (1 mM) and further grown for 18 h at 18 °C.

*H*_*6*_-*PEX5AzF* was co-expressed with pEVOL-*p*AzF, a vector encoding the orthogonal *p*AzFRS/tRNA_CUA_ pair^56^ (kindly provided by P.G. Schultz, Addgene plasmid 31186) in *E. coli* BL21 (DE3) in 2 L of M9-minimal media containing appropriate antibiotics as described before^43^. Cultures were grown to an OD_600_ = 0.3–0.4 at 37 °C. The unnatural amino acid p-azidophenylalanine (AzF; Bachem) was added to a final concentration of 1 mM. Protein expression was induced with IPTG (1 mM) and 0.02% arabinose for 12 h at 28 °C.

*Strep-Ub-GyrA-CBD* was expressed in *E. coli* BL21 (DE3) in 8 L of YEP containing appropriate antibiotics. Cells were grown to an OD_600_ = 0.4–0.6 at 37 °C, induced with IPTG (0.4 mM) and further incubated for 4 h at 28 °C.

### H_6_-PEX5/H_6_-PEX5AZF purification

Pelleted cells were resuspended in HisA buffer (50 mM NaH_2_PO_4_, 300 mM NaCl, 10 mM imidazole, pH 8) (5–7 mL/g pellet) and lysed using an EmulsiFlex®-C3 system (Avestin). After centrifugation (25,000 g, 1 h, 4 °C) the filtered supernatant was loaded onto a 1 mL HisTrap™HP column (GE Healthcare) for one hour. The proteins were eluted using a linear gradient of HisB buffer (50 mM NaH_2_PO_4_, 300 mM NaCl, 500 mM imidazole, pH 8) and dialysed against click buffer (20 mM HEPES, 150 mM NaCl, pH 8). H_6_-PEX5 was further purified on a Superdex™ 200 HiLoad™ 16/60 column (GE Healthcare) using click buffer. The purity of the proteins was confirmed by SDS-PAGE.

### Strep-Ub-GyrA-CBD purification

*E. coli* BL21 (DE3) producing Strep-Ub-GyrA-CBD were lysed using a sonifier (Branson; 40% amplitude, 0.5 sec pulse on/off, 3 min total time) in chitin binding (CB) buffer (20 mM HEPES, 500 mM NaCl, 1 mM EDTA, pH 8.2). After centrifugation (25,000 g, 1 h, 4 °C) the filtered supernatant was incubated with chitin resin (New England Biolabs) for 2 h at 4 °C. After washing, thiolysis and aminolysis were performed on the column by adding CB buffer supplemented with 150 mM MESNA and 750 mM propargylamine (1.25 mL CB buffer/1 mL resin). Following incubation for 36–40 h at 4 °C, the cleaved Strep-Ub-alkyne was extensively dialysed against click buffer to remove the propargylamine and further purified on a Superdex™ 75 16/60 column (GE Healthcare). Alkyne generation was confirmed by electrospray ionization-mass spectrometry using a Shimadzu LCMS-2020. The purity of Strep-Ub-alkyne was confirmed by SDS-PAGE.

### Copper (I)-catalysed alkyne-azide cycloaddition (CuAAC) reaction

Strep-Ub-alkyne and H_6_-PEX5AzF (24 µM and 8 µM, respectively) were mixed in click buffer with 200 µM ascorbate, 50 µM tris(benzyltriazolylmethyl)amine (TBTA; in 1:4 DMSO/tBuOH), 250 µM SDS and 500 µM CuSO_4_ resulting in H_6_-PEX5-Ub-Strep. The reaction was incubated for 30 min at room temperature and stopped with EDTA (10 mM final concentration). After dialysis against HisA buffer, H_6_-PEX5-Ub-Strep was first purified using a 1 mL HisTrap™HP column as described above. The eluted protein was directly loaded onto a 1 mL Strep-Tactin® Superflow® high capacity cartridge (IBA) using StrepA buffer (20 mM Tris, 150 mM NaCl, 1 mM EDTA, pH 8). H_6_-PEX5-Ub-Strep was eluted using a linear gradient of StrepB buffer (20 mM Tris, 150 mM NaCl, 1 mM EDTA, 5 mM desthiobiotin, pH 8). Purified H_6_-PEX5-Ub-Strep was dialysed against click buffer. The success of the click reaction and purification of H_6_-PEX5-Ub-Strep was confirmed by SDS-PAGE.

### Capillary electroporation of human fibroblasts

PEX5-deficient human fibroblast cells (PDB005T)^45^ were referred to as ΔPEX5 fibroblasts. Cells were cultured in Dulbecco’s modified Eagle medium (DMEM; Sigma) supplemented with 10% fetal calf serum, 2 mM glutamine and 50 mg/L gentamicin at 37 °C and 8.5% CO_2_. Capillary electroporation^57^ was carried out with a Neon™Transfection System (Life Technologies). Cells were washed twice with Hank’s Balanced Salt Solution (HBSS) and resuspended in electroporation buffer (250 mM sucrose, 1 mM MgCl_2_ in Dulbecco’s-PBS)^58^. Each 10 µL electroporation reaction containing 2 × 10^5^ ΔPEX5 fibroblasts and purified H_6_-PEX5 (4 µM), Strep-Ub-alkyne (4 µM) or H_6_-PEX5-Ub-Strep (8 µM) was pulsed at 1050 V (30 ms). To monitor the PTS1 import we added 66 ng pEGFP-PTS1 (pEB22.11, coding for EGFP with the C-terminal sequence RPPLHSKL) as reporter plasmid. After electroporation cells were resuspended in pre-warmed supplemented DMEM and sedimented at 200 g for 5 min to remove recombinant proteins. Cells were then seeded either into 6-well-plates prepared with coverslips for indirect immunofluorescence microscopy or into 25 cm^2^ culture flasks for immunoblot detection.

### Cytosol extraction from ΔPEX5 fibroblasts

For cytosol extraction four to seven 175 cm^2^ culture flasks of 90% confluent ΔPEX5 fibroblasts were harvested and resuspended in cytosol extraction buffer (20 mM HEPES, 150 mM NaCl, 5 mM imidazole, 20% glycerol, pH 7.4, protease inhibitor cocktail (PI) (Sigma; 1:200)). To isolate the post-nuclear supernatant (PNS), cells were lysed using a pre-cooled ball-bearing-homogenizer^59^, centrifuged (700 g, 10 min, 4 °C) and the pellet resuspended in cytosol extraction buffer. The centrifugation was repeated and the resulting two supernatants (PNS) were pooled and pelleted (100,000 g, 1 h, 2 °C) to separate the cytosol from the organelles. The cytosolic fraction was stored in aliquots at −80 °C.

### Immunoblot detection

For an SDS-PAGE gel either 20 µg of total protein after electroporation or 1.1% of bound and 2.8% of unbound fractions after pull-down binding assays were separated and transferred onto a nitrocellulose membrane (Roth) using a semidry blotting system (Biorad). α-PEX5 (GDA2, rabbit polyclonal) was diluted 1:5,000 in TBS-TX buffer (100 mM Tris, 100 mM NaCl, pH 7.4, 0.1% Triton X®100). To generate these PEX5 antibody pVS1 was expressed in *E. coli* BL21 (DE3) and recombinant protein was purified under native conditions as described^20^. The α-MAPK (α-mitogen activated protein kinase) antibody (QIAexpress® Tag 100, Qiagen, against the epitope of MAP kinase 2 (EETARFQPGYRS); mouse monoclonal) was diluted 1:2,000 in TBS-Tw buffer (100 mM Tris, 100 mM NaCl, pH 7.4, 0.02% Tween®20). After using the enhanced chemiluminescence Western Blot substrate (Thermo Scientific) the blot was exposed to film (Hyperfilm; Amersham) for different times and developed.

### Immunofluorescence microscopy

Cells on coverslips were stained for immunofluorescence microscopy as described before^60^. As primary antibodies α-thiolase (ACAA1) (rabbit polyclonal, dilution 1:100; a kind gift of Nancy E. Braverman), α-PMP70 (ABCD3) (sheep 1:100; a kind gift of Stephen J. Gould), α- PEX14^61^ (rabbit polyclonal 1:400) or α-AFP (mouse monoclonal (3E6), 1:100) (QBiogen) were used. The secondary antibodies (donkey) α-mouse or α-rabbit conjugated to Alexa-488 or Alexa-594 were purchased from Life Technologies and were used in a dilution of 1:300 and 1:200, respectively.

The stained cells were analysed using a Zeiss Axiovert 200 M fluorescence microscope equipped with a Plan-Apochromat 63x/1.4 oil objective and AxioVision 4.8 software.

### *In vitro* synthesis of proteins and autoradiography

The *in vitro* transcription and translation (TNT) experiments were performed with the TNT® Quick Coupled Transcription/Translation System (Promega) according to manufacturer’s instruction. The translation products from the rabbit reticulocyte lysate (RRL) were labelled with ^35^S-methionine (35 mCi/mL; Hartmann Analytic). The amounts of protein synthesised in our reactions were not determined but yields of 2–6 ng of protein/microliter of lysate are common according to the manufacturers information. The reactions were carried out for two hours at 30 °C. Generation of PEX26-myc (referred to PEX26) was performed together with the chaperone PEX19^62^ (pCS3). For this, both plasmids were first transcribed/translated individually for 15 min and then mixed for the rest of time. PEX1 and PEX6^63^ (pTY03) were either synthesised alone or mixed together after 45 min (then referred to as PEX1/PEX6). All TNT and pull-down samples were heated for 5 min at 80 °C and analysed on a 10% SDS-PAGE gel. An aliquot of 0.075 µL to 2.5 µL TNT sample was used. The gels were soaked in 0.5 M Na-salicylate for two times 30 min, dried and subjected to autoradiography (Carestream BioMax MR, Sigma) for 2–10 days. The transcripts for PTS2-CAT^6^, PTS2-GFP^64^ (pJM205) and pre-SCP2^65^ were generated by PCR to include a T7 promotor. PTS2-CAT was amplified using the forward and reverse primer pairs GD532 (5′-GAATTTAATACGACTCACTATAGGGAGGATCCACCATGCATCGGCTGCAGGTA-3′) and GD530 (5′-GGTATTCCATATGTTACGCCCCGCCCTG-3′); PTS2-GFP with GD532 and GD531 (5′-GGTATTCCATATGTTACTTGTACAGCTCGTCCATG-3′) and pre-SCP2 with GD546 (5′-GAATTTAATACGACTCACTATAGGGAGGATCCACCAT GGGTTTTCCGGAAGCC-3′) and GD547 (5′-GGTATTCCATATGTCAGAGCTTAG CGTTGCC-3′). The TNT reactions with PCR products (1.2 µg DNA for pre-SCP2 and 0.80 µg for PTS2-CAT and PTS2-GFP) were supplemented with T7 TNT PCR enhancer.

### Pull-down binding assays with Ni-beads

Magnetic Ni-beads (His Mag Sepharose™ Ni; GE Healthcare; 40 µL per reaction) were coupled with 7 µg of purified H_6_-PEX5 and 14 µg H_6_-PEX5-Ub-Strep or without protein (control) in buffer A (cytosol extraction buffer with 0.02% Tween®20) for 45 min at 4 °C. Protein concentrations of purified fractions were estimated at 280 nm using the respective extinction coefficients (Expasy). As this was difficult for H_6_-PEX5-Ub-Strep, due to the unusual linkage, the amounts coupled to the beads were adjusted to obtain equivalent band intensities on a Coomassie-stained SDS-PAGE gel. The coupled Ni-beads were incubated with the radiolabelled translation products (10 µL reaction samples of PEX14^61^, 20 µL of PEX1, PEX6, PEX19/PEX26, PEX13, pre-thiolase-myc^66^ (referred to as thiolase), myc-PEX7^14^ (referred to as PEX7; pEB13.10), PTS2-CAT^6^, PTS2-GFP^64^ (pJM205), PTS1-GFP (pSM102)^64^ and pre-SCP2^65^ and 40 µL of PEX1/PEX6) either in buffer A or in ΔPEX5 fibroblast cytosol supplemented with 2 mM ATP (Sigma) or 2 mM ATPγS (Sigma) and 4 mM MgCl_2_ (150 µL total incubation volume) for 2 h at 30 °C. If necessary, the reactions were filled up with RRL to have the same amount of RRL in each sample. After incubation, unbound fractions were removed and the Ni-beads were washed four times prior to elution with 30 µL buffer B (20 mM HEPES, 150 mM NaCl, 200 mM imidazole, 20% glycerol, pH 7.4, PI (1:200) and 0.02% Tween®20) for 10 min.

### Pull-down binding assays with myc-beads

Magnetic myc-beads (myc-Trap®_MA; Chromotek; 20 µL per reaction) were used according to manufacturer’s instruction. The radiolabelled translation products (4 µL PHYH-myc^67^, 10 µL PEX14, 20 µL of PEX1, PEX6 and PEX13 and 40 µL of PEX26-myc/PEX19) were incubated with myc-beads in buffer C (20 mM HEPES, 150 mM NaCl, 20% glycerol, pH 7.4, PI (1:200) and 0.02% Tween®20) containing 2 mM ATP and 4 mM MgCl_2_ as described above under ‘Pull-down binding assays with Ni-beads’. The elution was performed with 30 µL buffer C containing 5 µg myc-peptide (Chromotek).

### Pull-down binding assays with Strep-beads

Magnetic Strep-beads (MagStrep “type3” XT beads; IBA; 40 µL per reaction) were coupled with purified 4.6 µg Strep-Ub-alkyne and 14 µg H_6_-PEX5-Ub-Strep or 14 µg H_6_-BRD4-GFP-Strep (control) in buffer C for 45 minutes at 4 °C. The unrelated control protein H_6_-BRD4-GFP-Strep (containing the bromodomain4(1)) was expressed from pET28a-BRD4(1)-TagGFP in *E. coli* BL21 (DE3) and purified by NTA-chromatography. The radiolabelled translation products (20 µL reaction samples of PEX1, PEX6, PEX19/PEX26) were incubated with Strep-beads in buffer C supplemented with 2 mM ATP and 4 mM MgCl_2,_ as described above under ‘Pull-down binding assays with Ni-beads’. The bound proteins were eluted with 30 µL buffer C containing 50 mM biotin.

### *In vitro* import assay

The principles of the PTS2-centered import assay including the preparation of mouse liver PNS (post nuclear supernatant) have been described before^47^. Briefly, 500 µg of PNS were incubated in 100 µL import buffer (250 mM sucrose, 50 mM KCl, 20 mM MOPS-KOH (pH 7.4), 3 mM MgCl_2_) supplemented with 3 mM ATP and the TNT ^35^S-lysate mix for 30 min at 37 °C or at 0 °C (control). The TNT ^35^S-lysate mix contained 0.37 µL of the PTS2 protein ^35^S-pre-thiolase, 0.67 µL ^35^S-PEX7 and 0.5 µL ^35^S-PEX5^11^ (pGD106), respectively. In addition, H_6_-PEX5 (0.96 µM) or H_6_-PEX5-Ub-Strep (0.84 µM and 1.69 µM) were added in some reactions. Proteinase K (PK; Sigma) was added in PK buffer (20 mM MOPS-KOH (pH 7.2)) to a final concentration of 400 µg/mL, incubated for 40 min at 0 °C and stopped with PMSF (500 µg/mL final concentration). The samples were filled up to 1 mL in SEMK buffer (250 mM sucrose, 1 mM EDTA (pH 8.0), 20 mM MOPS-KOH (pH 7.2), 80 mM KCl, and centrifuged (11,300 g, 15 min, 4 °C). The pellet was precipitated with 1 ml TCA (10%) washed with acetone, separated by SDS-PAGE and transferred onto a nitrocellulose membrane (Roth) using a semidry blotting system (Biorad). For the SDS gel 60% of the samples and 0.075 µL of the TNT reactions were used. The dried membrane was exposed to autoradiography for six to eight days.

## Electronic supplementary material

Supplementary Information

## Data Availability

No datasets were generated or analysed during the current study.
