# Supplementary material for: Chemically monoubiquitinated PEX5 binds to the components of the peroxisomal docking and export machinery
Source: Sci Rep. 2018 Oct 30;8:16014. doi: 10.1038/s41598-018-34200-5 (PMC6207756; doi:10.1038/s41598-018-34200-5)
Supplement: Supplementary file 1 — Supplementary Information [file 41598_2018_34200_MOESM1_ESM.pdf]

Supplementary Information

Chemically monoubiquitinated PEX5 binds to the components of the peroxisomal docking and export machinery

Vera Hagmann[1], Stefanie Sommer[2], Patrick Fabian[1], Jan Bierlmeier[1], Nadine van Treel[2], Henning D. Mootz[2], Dirk Schwarzer[1], Jorge E. Azevedo[3], Gabriele Dodt[1*]

[1]Interfakultäres Institut für Biochemie (IFIB), Universität Tübingen, Tübingen, Germany. [2]Institut für Biochemie, Universität Münster, Münster, Germany. [3]Instituto de Investigação e Inovação em Saúde (I3S), Instituto de Biologia Molecular e Celular (IBMC), and Instituto de Ciências Biomédicas Abel Salazar (ICBAS), University of Porto, Porto, Portugal.
[*]Correspondence and requests for materials should be addressed to G.D. (email: gabriele.dodt@uni-tuebingen.de)

a)

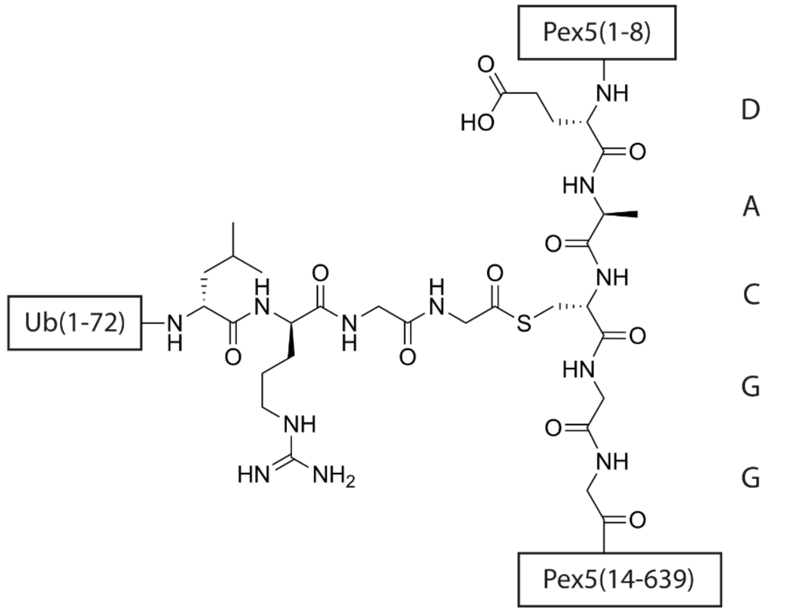

b)

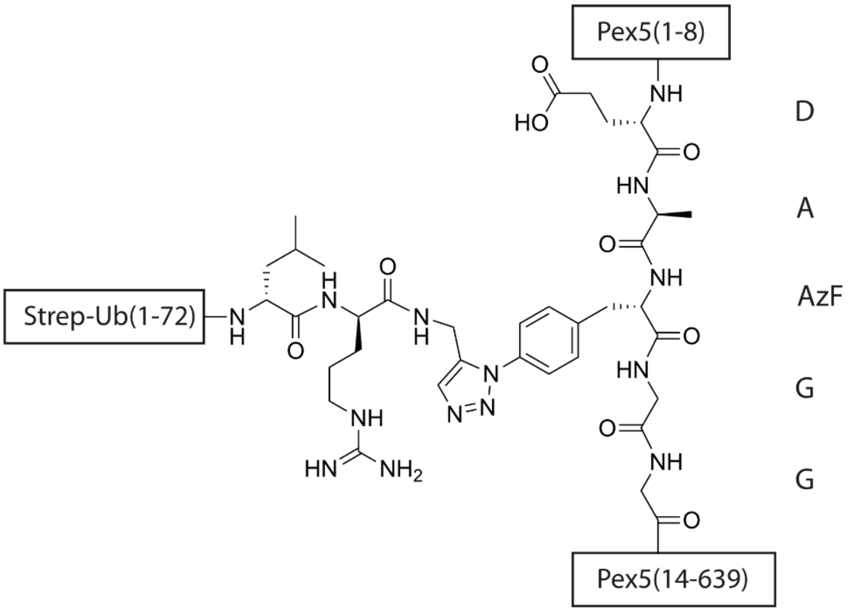

Supplementary Figure S1. Comparison between the natural linkage of PEX5 with ubiquitin and the linkage of recombinant H_6-PEX5-Ub-Strep. (a) Thioester between the C11 of PEX5 and the C-terminus of ubiquitin. (b) 1,2,3-Triazole linkage between PEX5C11AzF and the C-terminus of ubiquitin. Note that the ubiquitin moiety in this fusion protein lacks the last two glycines in order to approximate the native spacing between PEX5 and ubiquitin.

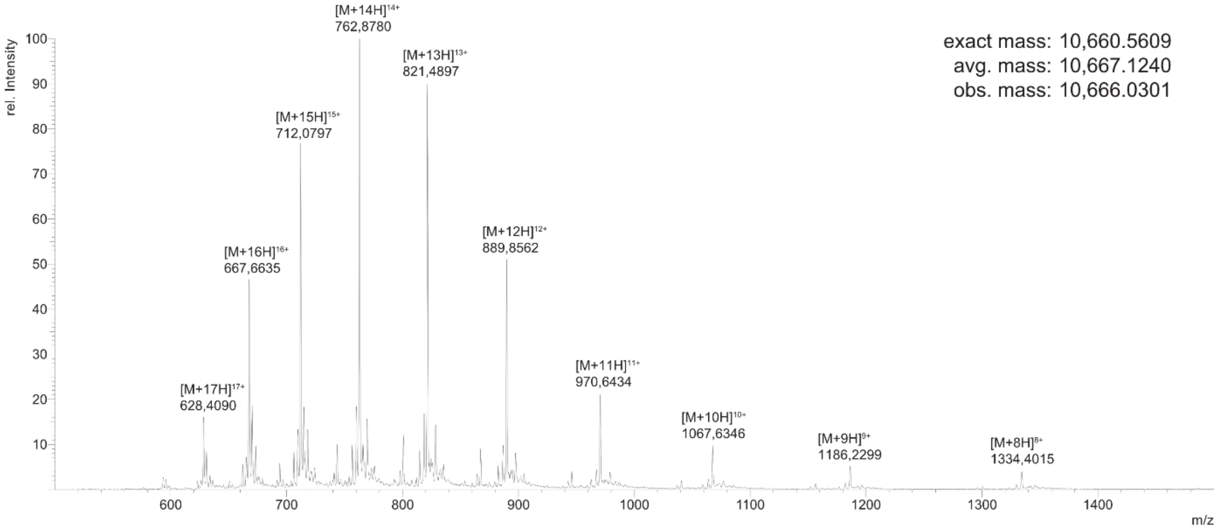

Supplementary Figure S2. Mass spectrum of Strep-Ub-alkyne. The observed mass (obs. mass) was obtained by spectral deconvolution with the Multi Charged Ion Analysis software package. The monoisotopic mass (exact mass) and molecular weight (avg. mass) of Strep-Ub-alkyne was calculated by the ChemDraw software package. The experiment was performed four times with similar results.

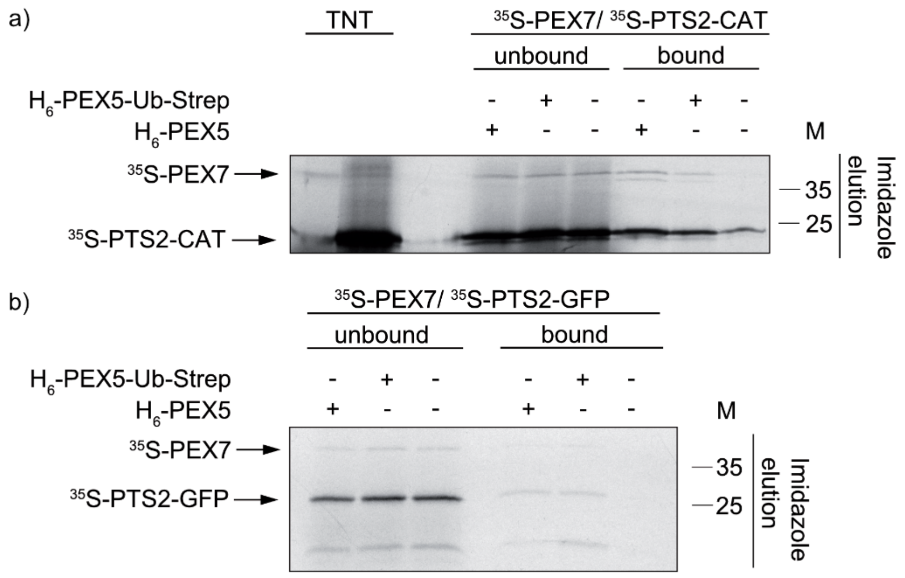

Supplementary Figure S3. Functional studies of recombinant H_6-PEX5-Ub-Strep with PTS2 proteins *in vitro.* Pull-down assay with magnetic Ni-beads that were coupled with purified H_6-PEX5 and H_6-PEX5-Ub-Strep, respectively or without protein (control). After incubating the beads with *in vitro* synthesised (a) ^{35}S-PEX7 and ^{35}S-PTS2-CAT or (b) ^{35}S-PEX7 and ^{35}S-PTS2-GFP, buffer A and ATP, the samples were eluted with imidazole. The bound (50%) and the unbound (10%) fractions as well as the input (TNT) were analysed by SDS-PAGE/autoradiographic detection. The numbers to the right indicate the molecular mass (M) of proteins in kDa. The full-length versions of the gels are presented in Supplementary Figure S6. The data for both experiments are representative for three replicates, each.

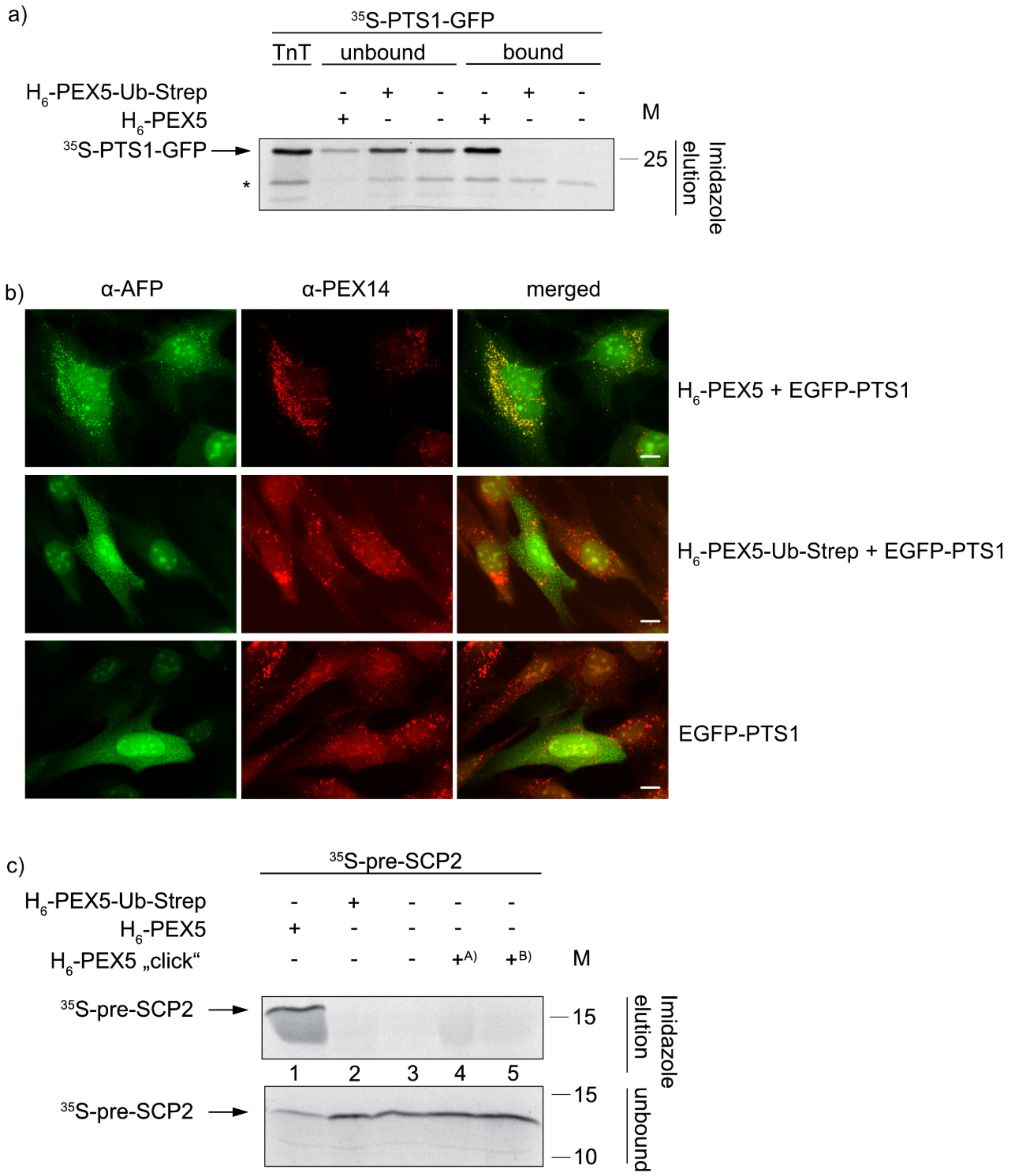

Supplementary Figure S4. Functional studies of recombinant H_6-PEX5-Ub-Strep with PTS1 proteins *in vivo* and *in vitro*. (a) Pull-down assay with magnetic Ni-beads that were coupled with purified H_6-PEX5 and H_6-PEX5-Ub-Strep, respectively or without protein (control). After incubating the beads with *in vitro* synthesised ^{35}S-PTS1-GFP, buffer A and ATP, the samples were eluted with imidazole. The bound (50%) and the unbound (10%) fractions as well as the input (TNT) were analysed by SDS-PAGE/autoradiographic detection. The experiment was performed twice with similar results. (*) The asterisk to the left represents an unspecific

translation product. (b) Human ΔPEX5 fibroblasts were electroporated with purified H_6-PEX5, H_6-PEX5-Ub-Strep and the reporter plasmid pEGFP-PTS1 (control), respectively. On the second day, an immunofluorescent staining was performed with α-PEX14 (Alexa 594) and α-AFP (directed against EGFP) (Alexa 488). Only H_6-PEX5 could import EGFP-PTS1 into peroxisomes which is colocalized with PEX14 (14% complementation rate), while no import was detected with H_6-PEX5-Ub-Strep or with pEGFP-PTS1 alone (control). Complementation rates were calculated by counting punctate EGFP-PTS1 positive cells in relation to all transfected cells. Cells that are not complemented display a cytosolic staining of EGFP-PTS1. At least 500 cells were counted for each electroporation. The experiment was performed twice with H_6-PEX5-Ub-Strep and four times with H_6-PEX5. The scale bar represents 20 µm. (c) Pull-down assay with magnetic Ni-beads that were coupled with purified H_6-PEX5 and H_6-PEX5-Ub-Strep, respectively and with H_6-PEX5 subjected to the conditions used for the CuAAC reaction (see methods, however omitting the SDS) referred to as H_6-PEX5 "click". To investigate the binding of the PTS1 protein ^{35}S-preSCP2, Ni-beads were incubated with *in vitro* synthesised ^{35}S-preSCP2, buffer A and ATP. The samples were eluted with imidazole. A) and B) refer to 14 µg or 28 µg of H_6-PEX5 "click", respectively. The experiment was performed once. The numbers to the right indicate the molecular mass (M) of proteins in kDa (a and c). The full-length versions of all gels in (a) and (c) are presented in Supplementary Figure S6.

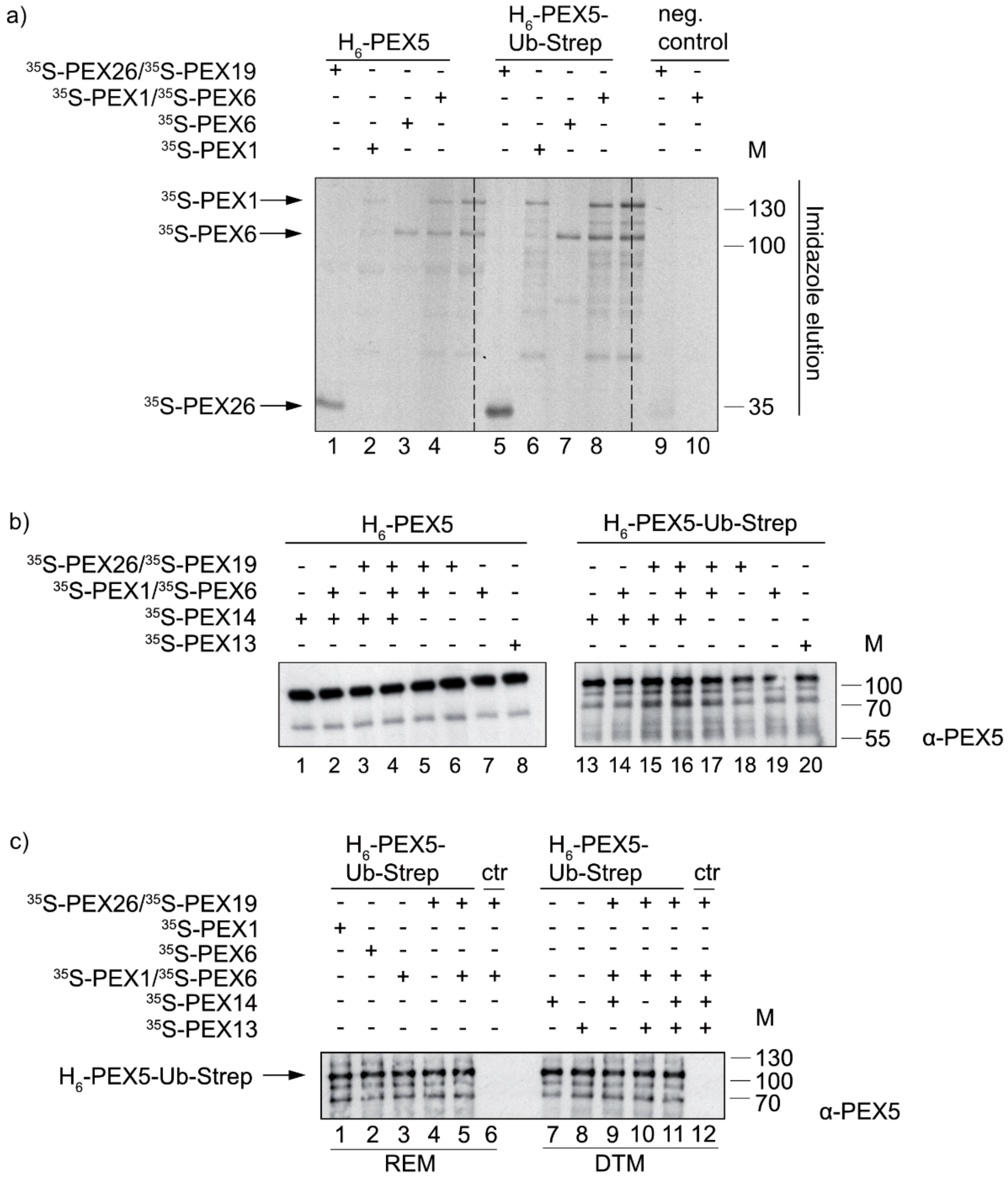

Supplementary Figure S5. H_6-PEX5 and H_6-PEX5-Ub-Strep interact with the peroxisomal export machinery. (a) Pull-down assay with magnetic Ni-beads that were prior coupled with purified H_6-PEX5 and H_6-PEX5-Ub-Strep or used uncoupled (control). *In vitro* synthesised ^{35}S-PEX1, ^{35}S-PEX6, ^{35}S-PEX1/^{35}S-PEX6 or ^{35}S-PEX26/^{35}S-PEX19 were added to the beads and incubated with cytosol from human ΔPEX5 fibroblasts and ATP. The samples were eluted with imidazole. The eluates (50%) were analysed by SDS-PAGE/autoradiography. The dash lines indicate irrelevant lanes. The contrast of this gel was slightly enhanced. The experiment was performed two times with similar results. (b) The eluates (2.9%) of the pull-down assay in Figure 4a with cytosol and ATP were separated on SDS PAGE, blotted to

nitrocellulose and analysed by immunoblot detection with α-PEX5. (c) The eluates (2.9%) of the pull-down assay in Figure 4b with buffer and ATPγS were also analysed by immunoblot detection with α-PEX5. The numbers to the right indicate the molecular mass (M) of proteins in kDa. The full-length original gels are presented in Supplementary Figure S6.

uncropped Figure 1c

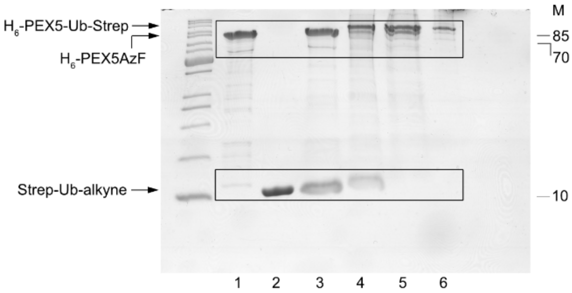

uncropped Figure 2a

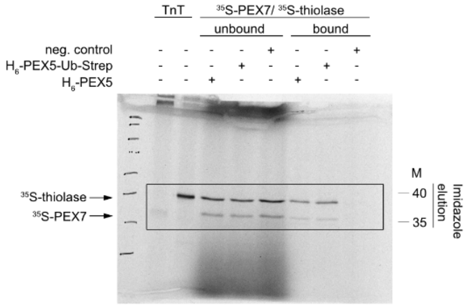

uncropped Figure 2b

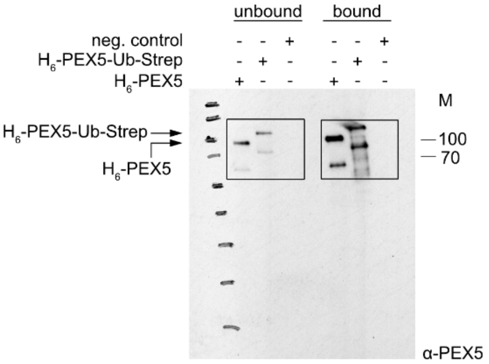

uncropped Figure 2d

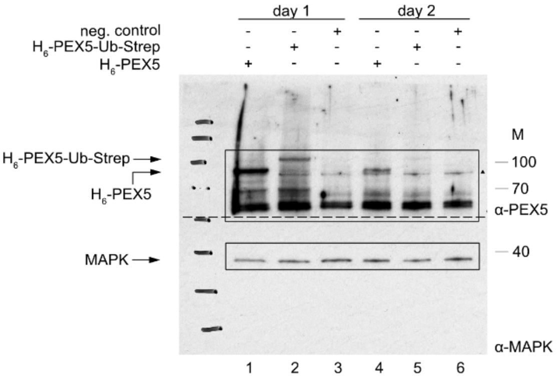

uncropped and unprocessed Figure 3

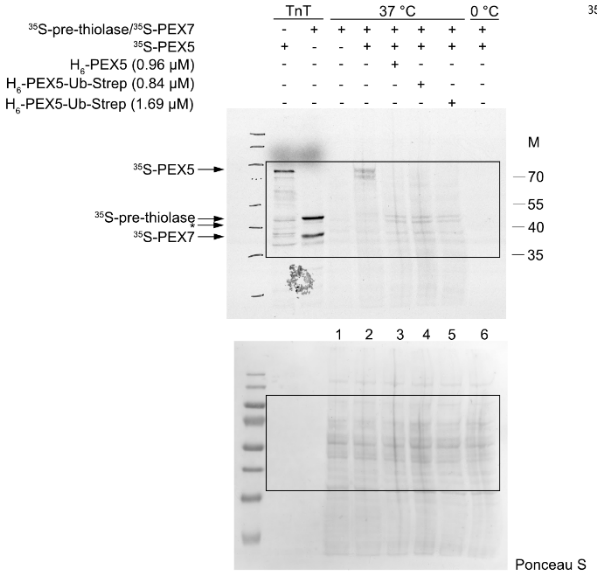

uncropped Figure 4c

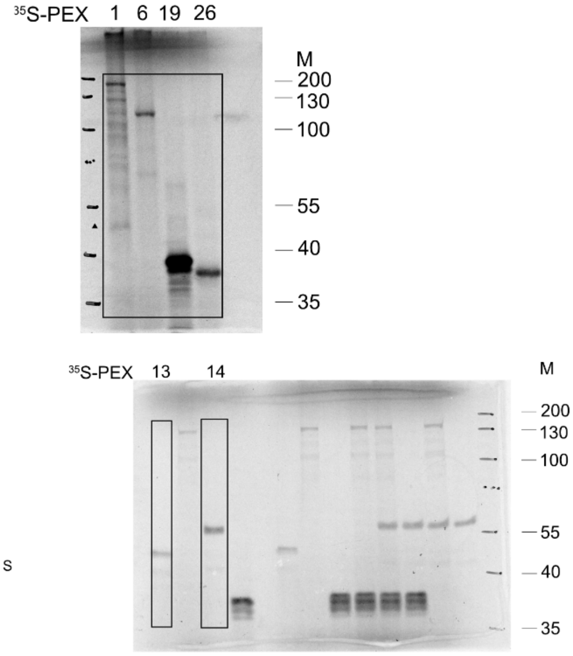

Supplementary Figure S6. Uncropped immunoblots and autographs. Boxes indicate the cropped panels.

uncropped Figure 5a

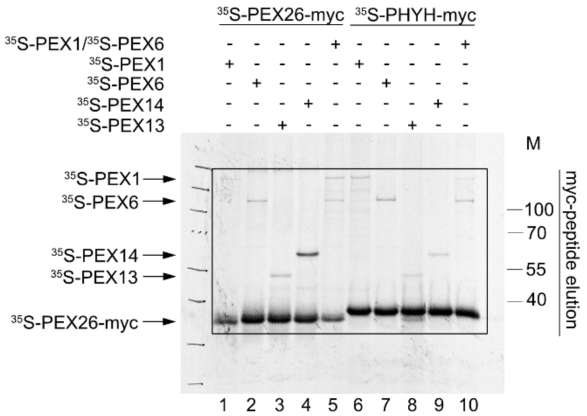

uncropped Figure 5b

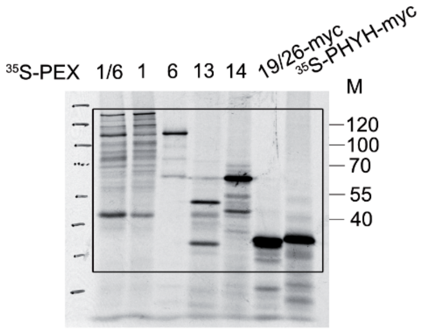

uncropped and unprocessed Figure 6

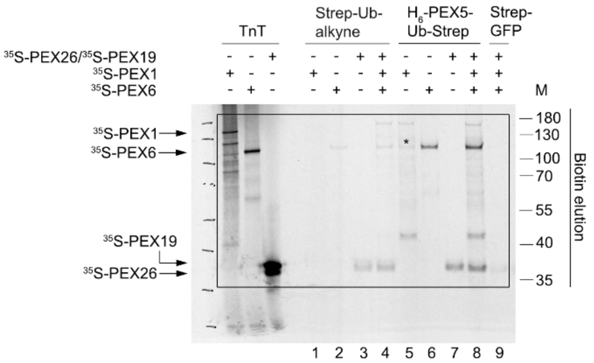

uncropped Supplementary Figure S3a

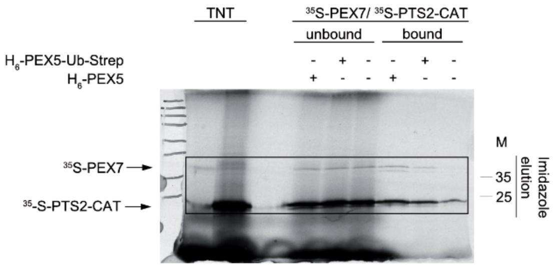

uncropped Supplementary Figure S3b

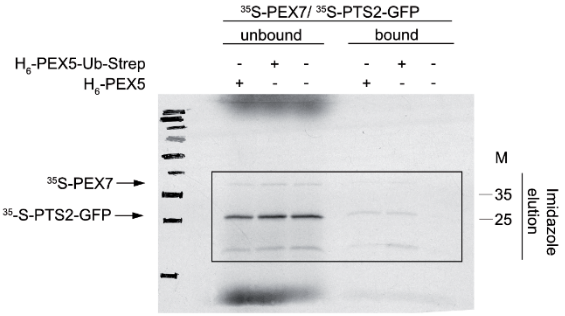

uncropped Supplementary Figure S4a

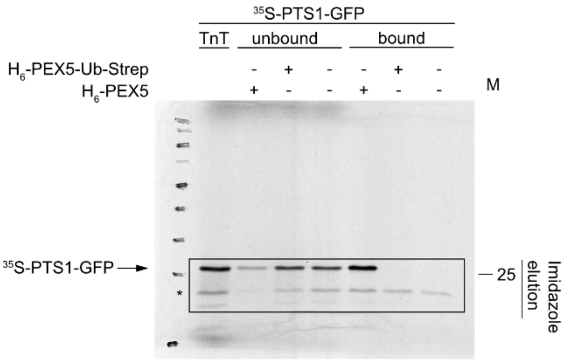

Continuation of Supplementary Figure S6.

uncropped Supplementary Figure S4c

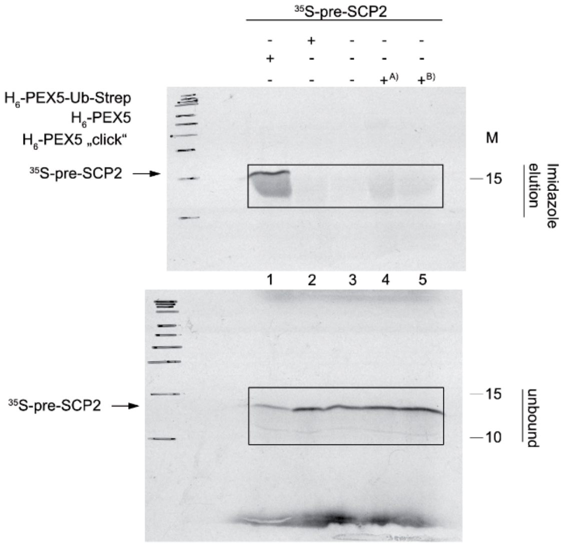

uncropped and unprocessed Supplementary Figure S5a

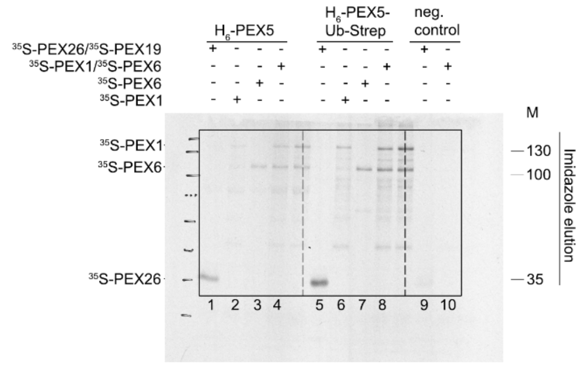

uncropped Supplementary Figure S5b

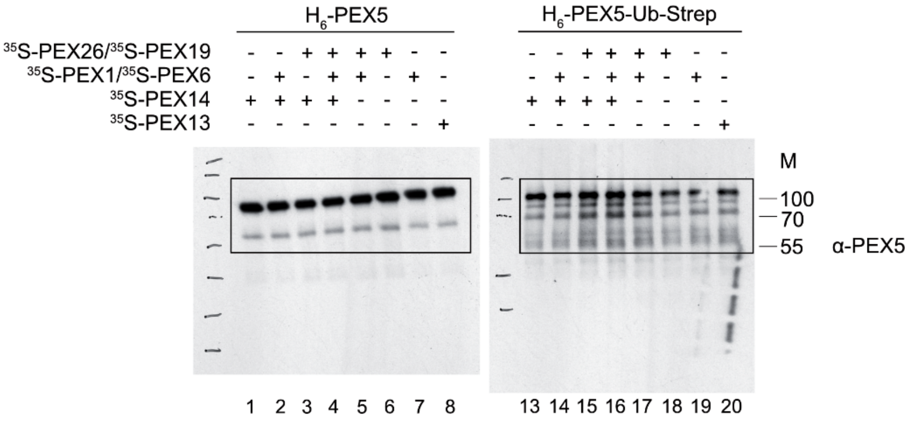

uncropped Supplementary Figure S5c

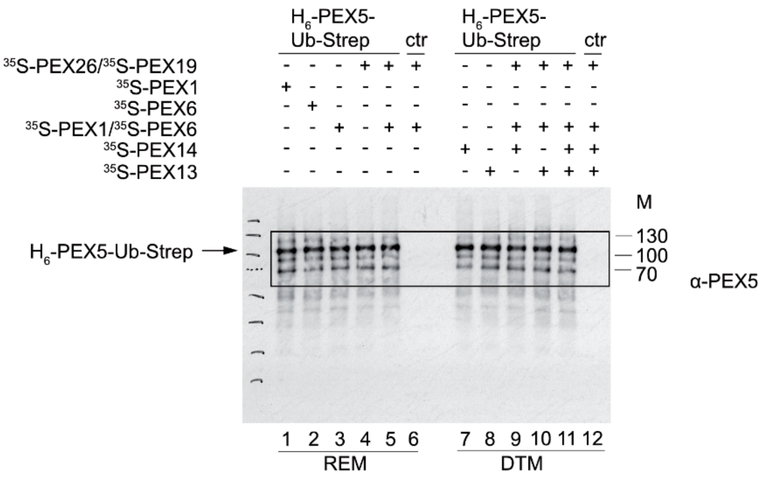

Continuation of Supplementary Figure S6

Supplementary methods

Electrospray Ionisation Mass Spectrometry

Monoubiquitinated PEX5, obtained by CuAAC, was analysed by LC-MS on an LCMS2020 System (Shimadzu, Neufahrn, Germany). The singe quadrupole mass analyzer was equipped with an electrospray ionisation (ESI) ion source and the LC was performed with a Kinetex® C18 column (2.6 µm, 2.1 x 100 mm, Phenomenex (Aschaffenburg, Germany)). H_6-PEX5-Ub-Strep was injected into the LC-MS system with formic acid (0.1 %; FA) in water (A) and 80 % acetonitrile, 0.1 % FA in water (B) as solvents. The protein was eluted and transferred into the MS-detector with a gradient of 5 – 95 % B within 12.75 min and a constant flow rate of 0.2 mL/min. The obtained MS spectra were analysed and deconvoluted by the Multi Charged Ion Analysis software package (Shimadzu, Neufahrn, Germany) using the following parameters: Smoothing: Savitzky-Golay 5 point; peak detection: Slope with 5 % threshold; deconvolution tolerance 0.2 Da; weight: Charge and intensity dependent. Exact and average masses of H_6-PEX5-Ub-Strep were calculated by the ChemDraw 16 software package (PerkinElmer Informatics, Cambridge, MA, USA).
